# Morphine Attenuates fNIRS Signal Associated With Painful Stimuli in the Medial Frontopolar Cortex (medial BA 10)

**DOI:** 10.3389/fnhum.2018.00394

**Published:** 2018-10-04

**Authors:** Ke Peng, Meryem A. Yücel, Sarah C. Steele, Edward A. Bittner, Christopher M. Aasted, Mark A. Hoeft, Arielle Lee, Edward E. George, David A. Boas, Lino Becerra, David Borsook

**Affiliations:** ^1^Center for Pain and the Brain, Harvard Medical School, Boston, MA, United States; ^2^Department of Anesthesiology, Critical Care and Pain Medicine, Boston Children’s Hospital and Harvard Medical School, Boston, MA, United States; ^3^MGH/HST Athinoula A. Martinos Center for Biomedical Imaging, Department of Radiology, Massachusetts General Hospital and Harvard Medical School, Boston, MA, United States; ^4^Neurophotonics Center, Boston University, Boston, MA, United States; ^5^Department of Anesthesia, Critical Care and Pain Medicine, Massachusetts General Hospital and Harvard Medical School, Boston, MA, United States

**Keywords:** analgesia, functional near infrared spectroscopy, morphine, nociception, pharamacokinetics, brodmann area 10

## Abstract

Functional near infrared spectroscopy (fNIRS) is a non-invasive optical imaging method that provides continuous measure of cortical brain functions. One application has been its use in the evaluation of pain. Previous studies have delineated a deoxygenation process associated with pain in the medial anterior prefrontal region, more specifically, the medial Brodmann Area 10 (BA 10). Such response to painful stimuli has been consistently observed in awake, sedated and anesthetized patients. In this study, we administered oral morphine (15 mg) or placebo to 14 healthy male volunteers with no history of pain or opioid abuse in a crossover double blind design, and performed fNIRS scans prior to and after the administration to assess the effect of morphine on the medial BA 10 pain signal. Morphine is the gold standard for inhibiting nociceptive processing, most well described for brain effects on sensory and emotional regions including the insula, the somatosensory cortex (the primary somatosensory cortex, S1, and the secondary somatosensory cortex, S2), and the anterior cingulate cortex (ACC). Our results showed an attenuation effect of morphine on the fNIRS-measured pain signal in the medial BA 10, as well as in the contralateral S1 (although observed in a smaller number of subjects). Notably, the extent of signal attenuation corresponded with the temporal profile of the reported plasma concentration for the drug. No clear attenuation by morphine on the medial BA 10 response to innocuous stimuli was observed. These results provide further evidence for the role of medial BA 10 in the processing of pain, and also suggest that fNIRS may be used as an objective measure of drug-brain profiles independent of subjective reports.

## Introduction

Previous neuroimaging studies have delineated various brain regions that exhibit a robust level of activation to pain at both subcortical (e.g., thalamus and amygdala) and cortical levels (e.g., insula and anterior cingulate cortex (ACC)) (Millan, 1999; Davis et al., 2005; Brooks and Tracey, 2007; Fuchs et al., 2014; Neugebauer, 2015). One area, the frontopolar (FP) cortex (also known as the anterior prefrontal cortex, aPFC), is a supramodal cortex that shares extensive connections with the sensory and emotion networks of the brain, and has emerged as an important brain region in the pain perception process (Peng et al., 2018a). Activation in the lateral portion of the FP cortex has been related to functions such as sensory/emotional assessment of pain conditions, pain modulation, empathy for pain and pain anticipation (Lorenz et al., 2003; Jantsch et al., 2005; Wiech et al., 2006; Peyron et al., 2007; Godinho et al., 2012; Palermo et al., 2015). Interestingly, besides activations, deactivations in response to noxious stimuli have also been reported, predominately in its medial portion (mainly the Brodmann Area 10, BA 10). Pain-induced BA 10 deactivation at both acute and chronic conditions has been observed across different imaging modalities such as functional magnetic resonance imaging (fMRI; Gündel et al., 2008; Lui et al., 2008; Kong et al., 2010; Tseng et al., 2010; Loggia et al., 2012), positron emission tomography (PET; Derbyshire et al., 1994; Hsieh et al., 1996; Vogt et al., 1996; Peyron et al., 1998; van Oudenhove et al., 2009; Yoon et al., 2014) and functional near infrared spectroscopy (fNIRS; Holper et al., 2014; Sakuma et al., 2014; Yücel et al., 2015; Aasted et al., 2016; Becerra et al., 2016; Kussman et al., 2016).

fNIRS employs near infrared lights to provide non-invasive, continuous measure of cortical hemodynamics in terms of oxygenated hemoglobin (HbO) and deoxygenated hemoglobin (HbR) concentration changes. However, fNIRS is limited by its imaging depth (~1–3 mm within the cortex) and susceptibility to hair contaminations. The location of medial BA 10 (below the forehead) is of particular interest in fNIRS studies due to the ease of access and the ability to achieve a high signal-to-noise ratio (SNR). By placing optodes on the forehead, previous fNIRS studies reported a significant deactivation in the medial portion of the BA 10 in various pain models, including cutaneous pain (Holper et al., 2014; Yücel et al., 2015), tooth pain (Sakuma et al., 2014) and visceral pain (Becerra et al., 2016; Kussman et al., 2016). Besides, the medial BA 10 response to painful events has been reproduced across awake (Yücel et al., 2015), sedated (Becerra et al., 2016) and anesthetized subjects (Kussman et al., 2016). Based on these observations, we suggested that the fNIRS-measured medial BA 10 signal might have the potential to be used as a brain marker involved in the complex nature of pain perception (Aasted et al., 2016; Peng et al., 2018b).

The deactivation of medial BA 10 following pain was often seen to be associated with other regions within the brain’s default mode network (DMN). The DMN, anchored by the medial PFC (including BA 10) and the posterior cingulate cortex (PCC), has been linked to task-independent “internal” functions (e.g., self-reference and interoception; Davey et al., 2016; Kleckner et al., 2017). The deactivation of the DMN by external-directed tasks has been therefore related to the switch of one’s attention from self to the outside stimulus (Prado and Weissman, 2011; Lin et al., 2015). As pain is intrinsically salient, the deactivation in the medial BA 10 (as part of the DMN deactivation) in response to noxious stimuli might potentially reflect an individual’s attention towards pain (Kucyi et al., 2013). Other studies proposed that the observed medial BA 10 deactivation might be a result of the neural activity suppression by the hyperactivity of amygdala (Ji et al., 2010), a core region that regulates the affective-motivational dimension of pain (Neugebauer, 2015). Despite the various proposed models, the explicit interpretation of the medial BA 10 signal following pain/nociception or analgesia has not been fully elucidated.

In this study, we sought to further understand the involvement of medial BA 10 in pain processing by evaluating the effects of a well-known analgesic, morphine, on its response to noxious stimulation with fNIRS. We measured the response to noxious stimuli in medial BA 10 using fNIRS before and after drug administration (a standard analgesic dose of 15 mg oral morphine in healthy volunteers) in a double blind, placebo-controlled design. Morphine is considered to be a “gold standard” opioid analgesic for nociceptive pain and is known to reduce the sensory and affective response to pain (Price et al., 1985). Indeed, previous animal and human studies have described the inhibitory effect of morphine on the activations of many cortical regions related to the sensory processing (e.g., the insula and somatosensory cortex) and/or emotional processing of pain (e.g., the ACC), see discussion “Effects of Morphine on BA 10 and S1 Responses to Noxious and Innocuous Stimuli” below. In this study, we hypothesized that morphine would also attenuate the fNIRS-measured deactivation in the medial BA 10 to painful experimental stimuli. The attenuation of pain signals in the medial BA 10 by a well-defined analgesic would: (a) provide further evidence for a role of BA 10 in the processing of pain and (b) support the potential use of the fNIRS-measured BA 10 signal to establish an objective measure of pain and analgesia.

## Materials and Methods

### Subjects

This study was approved by the Institutional Review Board (IRB) of the Massachusetts General Hospital, and conformed to the ethical standards for human experimentation as defined by the Helsinki Accord and the International Association for the Study of Pain. Fourteen healthy right-handed male subjects with no recent history of pain (age range: 22–37 years, mean ± standard deviation: 29 ± 5 years) completed this study. Written informed consent was obtained from each subject prior to experiments. Subjects with a history of allergy to opioids or opioid abuse, history of neurological trauma or psychiatric disorders, or who were unable to keep their head still for at least six consecutive minutes were excluded.

Each subject underwent two fNIRS scanning visits after the initial medical screening. In a double blind, randomized crossover design, subjects were given either immediate-release morphine (15 mg PO) or placebo (pills looked identical). An anesthesiologist was present during the entire procedure of both visits to monitor the possible adverse effects of morphine such as respiratory compromise. The washout time interval between the two visits for subjects varied from 3 weeks to 2 months depending on the availability of the subject and the research team.

### Experimental Procedures

**Figure 1:**
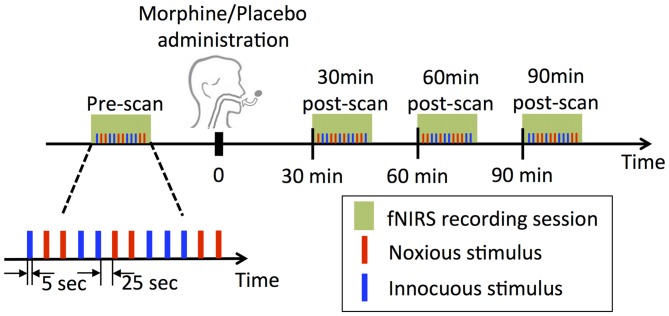
Experimental design. At each visit, four functional near infrared spectroscopy (fNIRS) sessions were performed, each of which contained a randomized sequence of six noxious and six innocuous stimuli. Note that subjects underwent two experimental sessions receiving either morphine or placebo first and then* vice versa* in a double blind fashion.

The experimental paradigm is summarized in Figure 1. In each visit of a subject, two subjective perception levels were first determined prior to the actual drug/placebo scans by applying electrical stimulations to subject’s left thumb with a 5 Hz electrical stimulator (Neurotron, MD, USA). The subject was asked to report at the scores of 3 and 7 over a 0–10 scale, with the 3/10 score being described as “*the subject should be strongly aware of the stimulus but shouldn’t perceive any pain*” (innocuous) and the 7/10 score being “*the subject should perceive much pain, but the pain should be tolerable without breath holding or any retreat actions*” (noxious) as in our previous studies (Peng et al., 2018b). The electrical intensities corresponding to the two perception levels were used in the following fNIRS scans.

Four fNIRS data acquisition sessions were conducted in each visit of a subject: the first scan was performed prior to the oral drug/placebo administration (the pre-scan), while the other three were conducted at exactly 30 min, 60 min and 90 min after the time when the drug/placebo was administrated (denoted as 30 min post-scan, 60 min post-scan and 90 min post-scan respectively, see Figure 1). Each scan lasted approximately 6 min, during which a total number of six innocuous stimuli and six noxious stimuli were delivered to the subject’s left thumb with a randomized sequence. The electrical stimulus (noxious or innocuous) was applied for 5 s and was followed by a 25 s resting period.

### Data Acquisition

**Figure 2:**
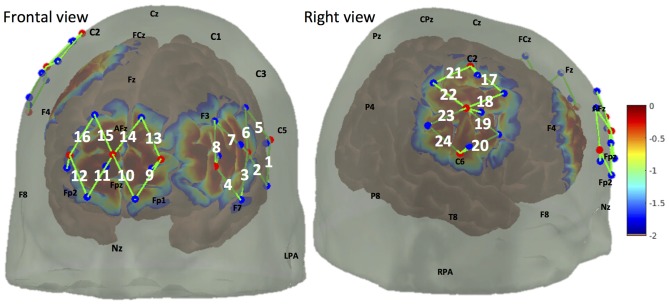
Optode arrangement over frontal lobe (medial brodmann area 10 (BA 10), left lateral prefrontal cortex (PFC)) and right somatosensory cortex. Optode arrangement and corresponding detection sensitivity are depicted in frontal view (left panel) and right view (right panel). Light emitters and detectors are shown with red dots and blue dots respectively. A NIRS channel consists of one light emitter and one light detector (shown by green lines and indexed in white). The sensitivity values are displayed on a logarithmic scale (with arbitrary units) showing the sensitivity of a cortical hemodynamic change being detected by the fNIRS measures.

We used a multichannel continuous wave fNIRS system (CW7 system, TechEn, MA, USA) to acquire optical data at 690 nm and 830 nm wavelengths. A total number of 24 30 mm fNIRS channels were mounted using nine light emitters and 12 light detectors (Figure 2). These channels mainly covered the medial portion of the FP cortex (medial BA 10), the right primary somatosensory cortex (S1), as well as part of the left lateral prefrontal cortex (lateral PFC), see “Cortical Responses in BA 10 and S1 to Noxious and Innocuous Stimuli” below for a discussion on the selection of covered brain regions. In addition to the 12 normal fNIRS detectors, nine short separation detectors placed 8 mm from adjacent light emitters were also installed to measure the signal changes from superficial layers (such as skin, scalp and skull).

### Data Pre-processing and Hemodynamic Response Function Estimation

The data pre-processing was carried out using the toolbox HOMER2 (Huppert et al., 2009) implemented in Matlab (Mathworks, MA, USA). Raw optical signals were first converted into optical density changes. We used the automatic detection function in HOMER2, which monitors the changes in signal amplitude and/or standard derivation of the time course to identify time periods that contain motion artifacts. Any stimulus that was within the interval of 20 s before an artifact to 5 s after the artifact was excluded from the analysis. No correction of motion artifact was performed (except for one subject, see below). The time courses were then reviewed manually. The optical density changes underwent a third-order Butterworth low-pass filter with a cutoff frequency of 0.5 Hz, and were then transformed into HbO and HbR concentration changes using the Modified Beer-Lambert Law. The concentration changes in total-hemoglobin (HbT) were obtained by a direct sum of the HbO and HbR changes (HbT = HbO + HbR). We estimated the hemodynamic response function (HRF) to noxious and innocuous stimuli with an ordinary least squares (OLS) approach, using a consecutive sequence of Gaussian functions as temporal basis functions (standard deviation = 1 s, separation of the mean = 1 s). For the time course of each channel, we included the short separation data that had the highest correlation as a static estimator to regress out the contamination of physiological signals (such as due to heartbeat or respiration). A third-order polynomial regressor was also applied in the model to remove the signal drifts. The physiological noise and drift-corrected HbO and HbR change time courses were reconstructed simultaneously from the OLS estimation (see Peng et al. (2018b) for details).

Following the estimation of the HRFs for each scan session (pre-scan and 30 min, 60 min, 90 min post-scans), the HRFs of one subject were normalized at each channel to the peak/nadir magnitude of the HRF obtained from the pre-scan. By normalizing the channel-wise HRFs to the pre-scan HRFs, we aimed to more intuitively view the percentage of attenuation of the HRFs in the post-scans and to mitigate the effect of the subject-specific difference in the shape and magnitude of the response in the comparison. This was achieved by identifying the highest value of a hemoglobin concentration increase (or the lowest value if a decrease was observed) in the pre-scan HRF within 2–15 s after the stimulus. We chose 2–15 s as the search window because; (a) all our stimuli lasted 5 s and (b) the delayed response of the hemodynamic change which usually occurs about 5–6 s after the underlying neuronal activity. At each channel, we then divided the four HRFs of a subject during a single visit by the identified pre-scan HRF peak/nadir magnitude to obtain the normalized set of HRFs.

### General Linear Model Analysis

For each scan session, we performed a standard general linear model (GLM) analysis to test the statistical significance of the detected hemodynamic response to the applied electrical stimuli. This was conducted with the Matlab toolbox nirs10 (Peng et al., 2014) developed based on SPM8 (Friston et al., 2006) and NIRS-SPM (Ye et al., 2009). Briefly, the GLM decomposes the reconstructed hemoglobin concentration change time course (see above) *Y* into a linear combination of the expected response to each time of stimuli *X*, a constant regressor and an error term, i.e., *Y* = *Xβ* + *ε*. The expected responses to the two types of electrical stimuli (noxious and innocuous) were computed by convolving the timing of the stimuli with a SPM8 canonical HRF. Before being passed to the GLM, the reconstructed time courses (see above) were high-pass filtered with a 4th-order Butterworth filter at 0.01 Hz and low-passed filtered using a filter with the shape of the canonical HRF (corresponding cutoff frequency ≈ 0.6 Hz; Ye et al., 2009). For each subject, the regression coefficients *β* corresponding to the stimuli were estimated at each channel, and were then interpolated to four two-dimensional views (i.e., frontal, right, left and dorsal) using an inhomogeneous interpolation kernel based on their locations on a brain MRI template. At each pixel of the four views, we conducted a two-tailed *t-test* on the interpolated regression coefficient *β*, testing the null hypothesis *H*_0_: *β* = 0, which is equivalent to ${H^{\prime }}_{0}$: the correlation between the recorded hemoglobin concentration change and the expected change is not statistically significant. For each scan session of each visit, the individual contrast maps of all the subjects were then pooled together to generate group-level contrast maps. This was carried out by using the precision-weighted averaging method (Ye et al., 2009) under a fixed-effect model (i.e., taking each subject as repetitive measures and assuming no inter-subject variance). The group-level *t*-contrast maps of each view were then corrected with a peak false discovery rate (pFDR)-based approach (Benjamini and Hochberg, 1995; Chumbley et al., 2010) to control the family-wise error.

## Results

### Data Included in the Analysis

No adverse effect of morphine was reported by any of the subjects during or after the recordings. The data of three subjects were discarded, as we were unable to obtain reliable measure of frontal brain activities. The data of the remaining 11 subjects (11/14, age range: 22–37 years, mean ± standard deviation: 30 ± 5 years) were included in the subsequent analysis, which contained a total number of 242 noxious stimuli and 239 innocuous stimuli during morphine visits and 221 noxious stimuli and 247 innocuous stimuli during placebo visits after exclusion for artifacts (Table 1). For one visit of a subject (the placebo visit of subject 1), we applied a targeted principal component analysis for motion correction (Yücel et al., 2014) because of the excessive artifacts observed in the dataset.

**Table 1:** Number of electrical stimulation trials included in the analysis.

#SUB	Morphine visit								Placebo visit							
	Pre		30 min		60 min		90 min		Pre		30 min		60 min		90 min	
	N	I	N	I	N	I	N	I	N	I	N	I	N	I	N	I
**1**	5	5	6	6	6	6	6	5	4	5	6	6	6	6	5	5
**2**	6	5	6	6	6	6	5	6	3	5	5	5	5	6	6	6
**3**	4	6	5	6	5	6	6	5	5	6	6	6	5	6	6	5
**4**	6	6	6	6	6	5	5	5	4	6	6	6	6	6	4	5
**5**	6	4	4	5	6	6	5	4	4	6	5	6	5	4	6	5
**6**	5	5	6	6	5	6	5	6	5	6	6	6	6	5	5	6
**7**	6	5	5	6	5	6	6	6	5	5	5	6	6	6	5	6
**8**	5	6	6	6	6	6	6	6	2	6	3	5	3	5	3	6
**9**	5	3	6	6	5	6	6	6	6	5	4	6	6	6	2	6
**10**	6	5	5	4	4	6	5	3	5	6	6	6	6	6	6	6
**11**	6	5	6	6	6	5	6	5	6	6	6	5	6	5	6	6
**Subtotal**	60	55	61	63	60	64	61	57	49	62	58	63	60	61	54	62

The visual analog scale (VAS) ratings of the noxious stimuli in the pre-scan and the post-scans were obtained from five randomly selected subjects (Supplementary Figure S1). We observed lower VAS ratings for electrical pain after the subjects took morphine comparing with the ratings prior to morphine administration, with the greatest reduction seen at 60 min post-morphine. However, we did not obtain statistical significant difference in the reductions of VAS ratings. On the other hand, no trend in the changes of the VAS rating of the perceived electrical pain was observed during the visits when oral placebo was given. These results of VAS ratings are further discussed in the section “Pain rating and BA 10 response” below.

### Response to Electrical Stimuli Over the Prefrontal Cortex

In this section, we focus on the HbO concentration changes to electrical stimuli. HbO response has often been observed to have a much higher SNR relative to HbR (Obrig et al., 2000; Yennu et al., 2016). In this study, an initial quality assurance analysis showed that the recorded HbO signals were more reliable in revealing the true shape of the hemodynamic response to external stimuli (results not shown). For HbR and HbT, the readers are referred to Supplementary Figures S2–S7.

#### Localized Hemodynamic Response to Electrical Stimuli in Normal Conditions

**Figure 3:**
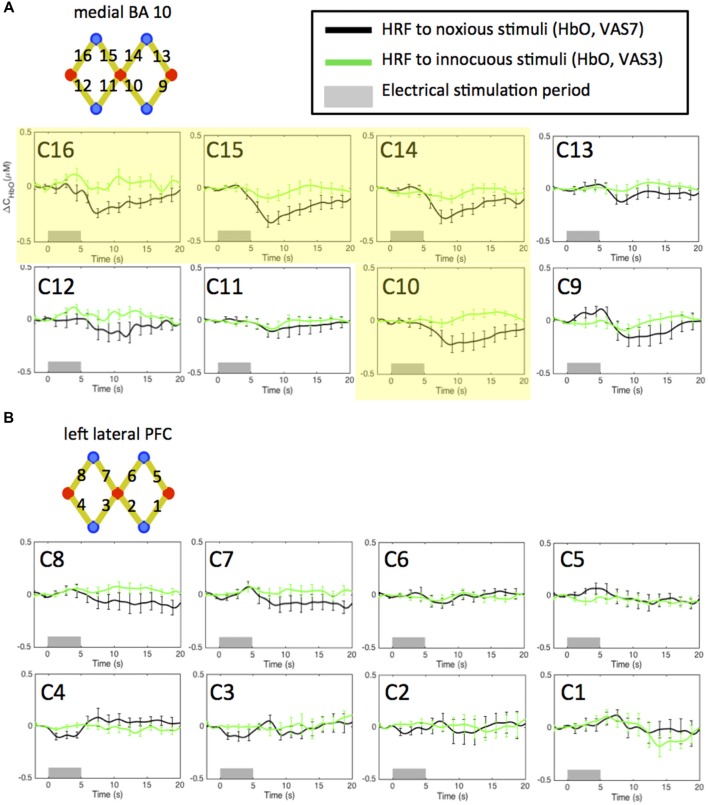
Estimated hemodynamic response functions (HRFs) in the pre-treatment scans across 11 subjects. Oxygenated hemoglobin (HbO) concentration changes to noxious and innocuous stimuli in the **(A)** medial BA 10 and **(B)** left lateral PFC in normal conditions. The pre-scans of the morphine visit and the placebo visit of each subject were combined. Channels showing statistically significant HbO decrease following noxious stimuli are highlighted in yellow. All error bars show the standard error of the mean.

In Figure 3, we plot the HRF of HbO concentration changes to noxious and innocuous electrical stimuli of the 11 subjects in normal conditions (i.e., the pre-scans, no morphine or placebo administered) acquired from the NIRS channels over the medial FP cortex (Figure 3A) and the left lateral PFC (Figure 3B). For each subject, the pre-scans of the morphine visit and the placebo visit were combined in this analysis. We observed significant HbO decreases in response to electrical stimuli mainly over the medial portion of the FP cortex (medial BA 10). Specifically, the nadir magnitudes of the HRFs to noxious stimuli from four channels (C10, 14, 15 and 16, highlighted in yellow in Figure 3A) in the pre-scans were seen to be statistically lower than zero (one-tailed *t*-test, *p* < 0.05). These channels were therefore included in the subsequent analysis to study the effect of morphine on medial BA 10 signals.

Deactivations in the medial BA 10 in response to innocuous stimuli were also observed in some of the channels. However, the responses were generally much weaker than those to noxious stimuli. These results are concordant with the findings reported in our previous studies (Yücel et al., 2015; Aasted et al., 2016).

On the other hand, we did not obtain any significant change of HbO concentration in the lateral PFC associated with either noxious or innocuous stimuli in the pre-scans (Figure 3B). The left lateral PFC results after the administration of morphine or placebo were therefore not presented in the following sections (please refer to Supplementary Figures S16–S21). We believe that the changes in the left lateral PFC following the administration of morphine or placebo depicted in the Supplementary Material may be less related to the pain perception process.

#### Effect of Oral Morphine on the HRF of Medial BA 10 Pain Signals

**Figure 4:**
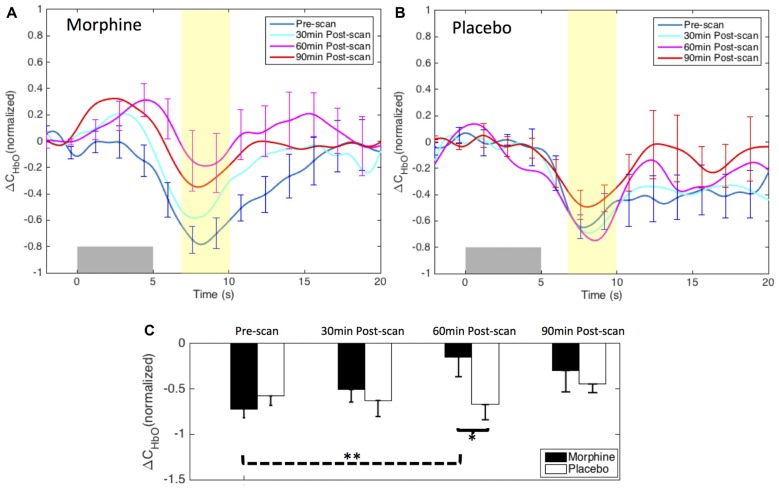
Morphine and Placebo effects on *noxious* stimuli in medial BA 10. Top panel: the normalized HRFs to noxious stimuli (VAS7) over the medial BA 10 during visits in which **(A)** oral morphine or **(B)** placebo was administered. Gray bars indicate the time period when noxious electrical stimuli were applied. Bottom panel: **(C)** bar plots of the averaged HRF magnitudes (from 7 s to 10 s post-stimulus) in each scan session. *P*-values showing statistically significant attenuation in the averaged magnitudes are marked in the figure, *p < 0.05, **p < 0.01. All error bars show the standard error of the mean. For image clarity, only the error bars of the pre-scan HRFs and the HRFs showing the most significant changes are depicted.

Figure 4 shows the HRFs of HbO concentration changes to noxious electrical stimuli (VAS 7) in the four fNIRS scan sessions (i.e., pre-scan, 30 min-, 60 min- and 90 min-post scan) of both visits. The HRFs of a single session were normalized to the peak/nadir magnitude of the pre-scan HRF, and were averaged first across channels then across the 11 subjects.

*Within-visit comparison*: during the visits when the subjects received oral morphine, we observed an attenuation of the HbO decrease associated with noxious stimuli with respect to HRF magnitudes when comparing the post-morphine scans with the pre-scan. Of note, such attenuation was seen to exhibit an inverted U-shape, i.e., reaching the maximum at 60 min after the administration of morphine (*p* = 0.006 for HRF magnitudes compared with those of the pre-scans from a one-tailed paired *t*-test). During the visits when placebo was given, the subjects showed clear HbO decreases over the medial BA 10 following noxious stimuli in all four scan sessions. No statistical significance was seen when we compared the nadir values of the pre-scan HRF to those of any of the three post-scan HRFs.

*Between-visit comparison*: we compared the HRF nadir magnitudes between the morphine visits and the placebo visits from corresponding scans. While no difference in the HRF magnitudes could be found in the pre-scans, medial BA 10 elicited much weaker deactivation responses to noxious stimuli at 30 min, 60 min and 90 min after the administration of oral morphine relative to placebo. Statistical significance was obtained between 60 min post-scans (*p* = 0.03).

*Post hoc statistical power analysis*: we conducted a *post hoc* analysis to estimate the statistical power of our approach to detect the effect of morphine using the software G*Power 3.1 (Faul et al., 2009). The effect size was calculated using the HRF magnitudes from 7 s to 10 s after the noxious stimulus onset (highlighted in yellow in Figure 4) of each scan. Assuming a significance level of 0.05, we obtained a power of 89% in the within-visit comparison (i.e., HRF magnitudes in pre-scan vs. 60 min post-scan during morphine visits), and a power of 62% in the between-visit comparison (i.e., HRF magnitudes in 60 min post-scan during morphine vs. placebo visits). These results implied that, with the current sample size, we had a moderate to good statistical power to detect meaningful effect of morphine on the BA 10 response to the electrical stimuli in the HRF analysis.

**Figure 5:**
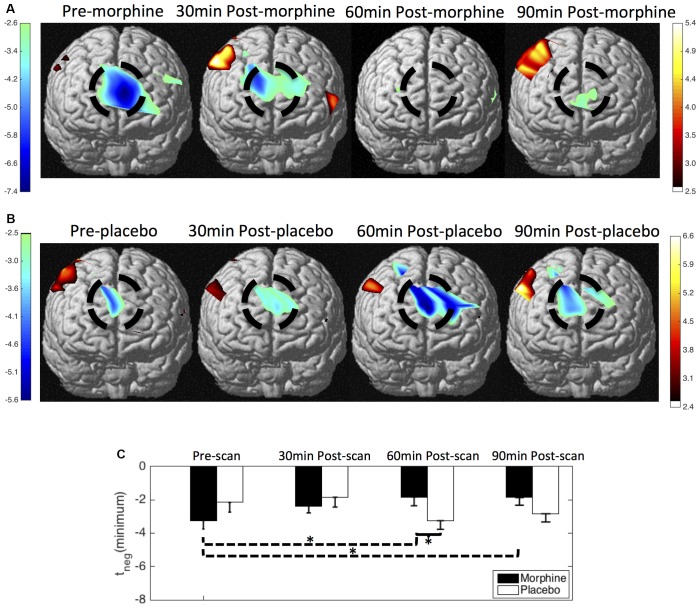
HbO contrast maps in the medial BA 10 associated with noxious stimuli (VAS7) generated from the general linear model (GLM) analysis. **(A)** Group-level statistical parametric maps of *t*-scores during the visits when the subjects received oral morphine, frontal view, peak false discovery rate (pFDR) corrected, *p* < 0.05. The located significant deactivations in the medial BA 10 are circled in black. **(B)** Group-level statistical parametric maps of *t*-scores during the visits when the subjects received oral placebo. **(C)** The minimum *t*-scores in the medial BA 10 area of each scan averaged across subjects. Statistically significant differences in the minimum *t*-scores between scans are marked in the figure, *p ≤ 0.05. Error bars depict the standard errors of the mean.

We show the contrast maps of *t*-scores from the GLM analysis of the HbO concentration changes of each scan in the visits when the subjects received morphine or placebo (Figure 5). Before morphine was administered, we observed a significant HbO deactivation over the medial BA 10 following noxious stimuli. The HbO deactivation in response to pain was attenuated at 30 min after morphine was given. At 60 min after morphine administration, no significant HbO deactivation cluster could be located in the medial BA 10. The HbO deactivation cluster reappeared at 90 min after morphine administration but with much lower absolute values of the *t*-scores and a much smaller spatial extent. Statistical significance in the minimum of *t*-scores of the medial BA 10 deactivations was seen between the pre-scans and 60 min post-scans (*p* = 0.03 from one-tailed Wilcoxon Signed Rank Test) and between the pre-scans and the 90 min post-scans (*p* = 0.02). Nonparametric tests were conducted to compare the minimum of the negative *t*-scores, as those *t*-values did not follow a Gaussian distribution. During the visits when the subjects received oral placebo, we were able to locate significant HbO deactivation clusters over the medial BA 10 in response to noxious stimuli in all of the four scans. While we observed no clear trend of attenuation of the deactivations with the administration of placebo, some changes in the *t*-scores and spatial extents with time were noticed.

When comparing the contrast maps between morphine and placebo visits in corresponding scans, we observed statistically lower negative *t*-scores in the medial BA 10 at 60 min after the administration of morphine than placebo (*p* = 0.05). These results from GLM analysis were largely consistent with those obtained from the HRF analysis described above.

**Figure 6:**
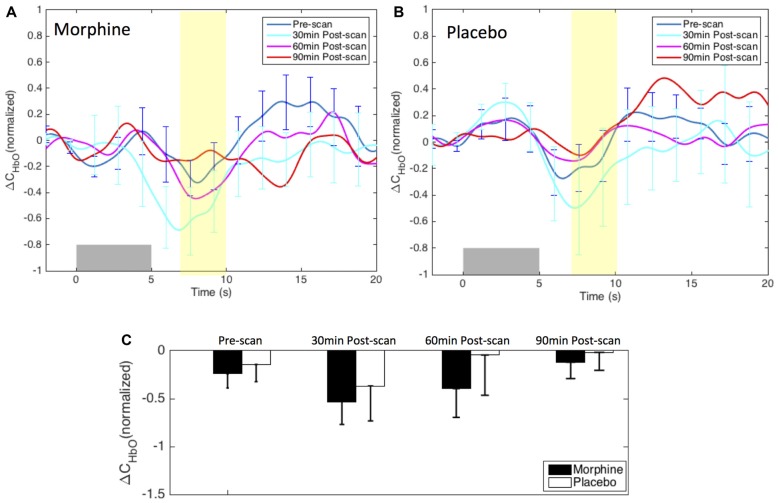
Morphine and placebo responses to *innocuous* stimuli in medial BA 10. Top panel: the HRFs to innocuous stimuli (VAS3) over the S1 during visits in which oral morphine **(A)** or placebo **(B)** was administered. Gray bars indicate the time period when innocuous electrical stimuli were applied. Bottom panel: **(C)** bar plots of the HRF magnitudes (from 7 s to 10 s after the onset of the stimulus) in each scan session. All error bars show the standard error of the mean. No statistical significant difference in the HRF nadir magnitudes between scans was seen.

We did not observe a clear modulation pattern of the medial BA 10 response to innocuous stimuli by the administration of either morphine or placebo (Figure 6). However, it may be of note that the post-scan HRFs showed an even more significant decrease in the HbO response to innocuous stimuli compared with the pre-scan HRF in both morphine and placebo visits, specifically at 30 min after drug/placebo administration. This trend might have similarly been observed in the BA 10 responses to noxious stimuli during placebo visits (see the placebo results in Figures 4, 5). We presume that these observations may be related to brain sensitization (i.e., increased response in nervous system to repeatedly applied stimuli), which potentially describes a learning process of the brain towards the applied stimulus in its first a few repetitions (Ursin, 2014). However, current results limited our ability to further explore the effect of sensitization. Indeed, the shapes of the medial BA 10 responses to innocuous stimuli were seen to be quite heterogeneous across scans and subjects, resulting in large error bars in the averaged HRFs and no statistical significance between any pair of scans. In the GLM analysis, the variability of the response to innocuous stimuli led to inconsistent HbO activation/deactivation clusters on the corrected *t*-scores in statistical contrast maps across scans, which generally didn’t allow meaningful interpretations with respect to morphine effects or sensitization. The contrast maps of *t*-scores from the GLM analysis of innocuous stimuli were therefore not presented in this section.

### Response to Electrical Stimuli Over the Primary Somatosensory Cortex

The involvement of the primary somatosensory cortex (S1) in the perception of pain has been reported in many previous studies. The S1 is included in the “lateral pain system” (regions to which pain information is projected through the lateral thalamic nuclei), and has been linked to the sensory-discriminative component of pain such as pain localization and intensity encoding (Hofbauer et al., 2001; Timmermann et al., 2001). Compared with the medial BA 10, fNIRS signals collected from the S1 are usually more susceptible to hair contaminations and motion artifacts, resulting in a lower SNR. Moreover, studies have shown that the activation in the S1 can easily be altered by cognitive factors including attention and previous pain experience (Bushnell et al., 1999). In this study, while our primary focus was on the fNIRS-measured medial BA 10 response, we located clear HbO increases in the contralateral S1 following noxious stimuli in normal conditions (i.e., in the pre-scan, without the administration of morphine or placebo) in 5 out of the 11 subjects (Subject #2, #3, #4, #6 and #11). The HbO data of these five subjects, which contained a total number of 93 noxious stimuli in the morphine visits and 91 noxious stimuli in the placebo visits (see Supplementary Table S1), were included to study the effect of oral morphine on the contralateral S1 response to pain. For HbR and HbT, please refer to Supplementary Figures S8–S11. For the results of innocuous stimuli, please refer to Supplementary Figures S12–S14.

**Figure 7:**
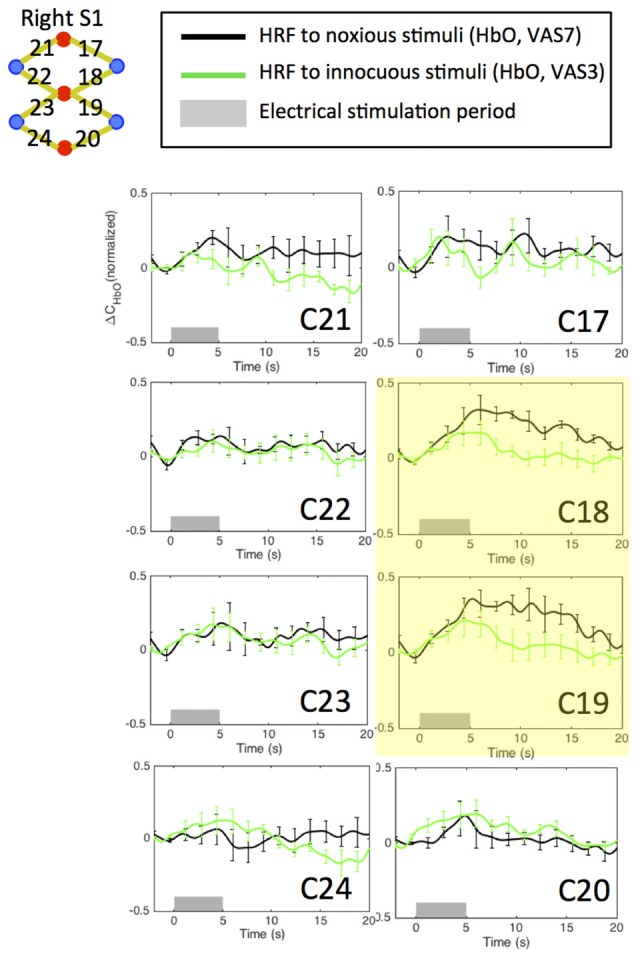
Estimated HRFs in the pre-treatment scans in the contralateral primary somatosensory cortex across five subjects. HbO concentration changes to noxious and innocuous stimuli in right S1 in normal conditions. For each subject, the pre-scans of the morphine visit and the placebo visit were combined in the analysis. The two channels showing strongest HbO increases following noxious stimuli are highlighted in yellow.

**Figure 8:**
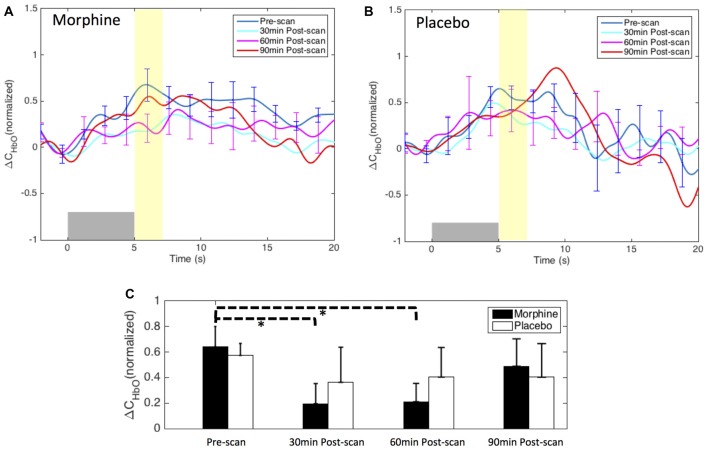
Morphine and placebo effects on the fNIRS-measured S1 responses to noxious stimuli. Top panel: the HRFs to noxious stimuli (VAS7) over the right S1 during visits in which oral morphine **(A)** or placebo **(B)** was administered. Gray bars indicate the time period when noxious electrical stimuli were applied. Bottom panel: **(C)** bar plots of the averaged HRF magnitudes (from 5 s to 7 s after the onset of the stimulus) in each scan session. *P*-values showing statistically significant attenuation in the averaged magnitudes are marked in the figure, *p < 0.05. All error bars show the standard error of the mean.

**Figure 9:**
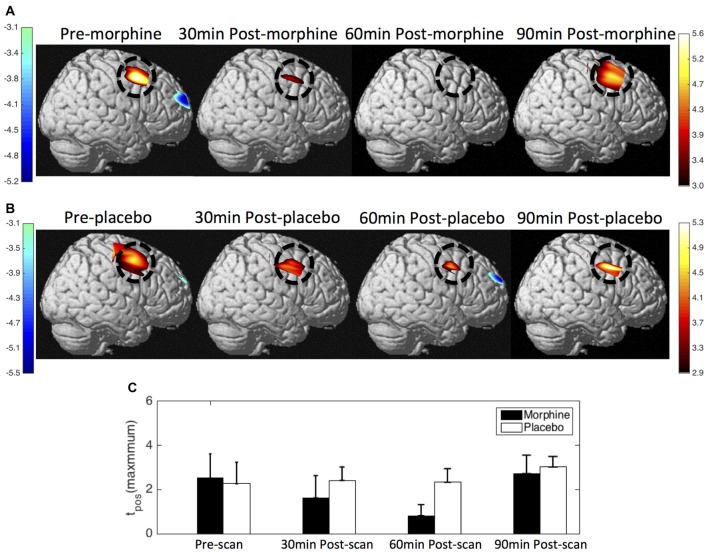
HbO contrast maps in the right S1 associated with noxious stimuli (VAS7) generated from the GLM analysis. **(A)** Group-level statistical parametric maps of *t*-scores during the visits when the subjects received oral morphine, right view, pFDR corrected, *p* < 0.05. In the cases that pFDR did not find a threshold, a fixed threshold of |t| > 3 was applied. The located significant activations in the S1 are circled in black. **(B)** Group-level statistical parametric maps of *t*-scores during the visits when the subjects received oral placebo. **(C)** The maximum *t*-scores in the right S1 area of each scan averaged across subjects. Error bars depict the standard errors of the mean.

An initial channel-wise HRF analysis revealed two channels (C18 and C19) that showed the most significant HbO response to pain (Figure 7). Figure 8 depicts the normalized HRFs of the four scans (pre-scan, 30 min, 60 min and 90 min post-scans) in the morphine visits and the placebo visits respectively. The estimated HRFs of each scan were first averaged across the two channels and then across the five subjects. Figure 9 shows the *t*-contrast maps of the HbO concentration changes associated with noxious stimuli in both visits generated from GLM analysis.

Both the HRF analysis and the contrast maps showed an attenuation of the HbO increase to noxious stimuli in the contralateral S1 at the group level with the administration of oral morphine. In the HRF analysis, we obtained statistical significance when comparing the HRF magnitudes (5–7 s after stimulus onset) between the pre-scan and the 30 min post-scan (*p* = 0.02 from one-tailed paired *t*-test) and between the pre-scan and the 60 min post-scan (*p* = 0.02). On the other hand, the administration of an oral placebo seemed to result in a much less significant attenuation of the S1 response to pain, with no statistical significance observed between scans.

## Discussion

### Summary of Results

To the best of our knowledge, this work is the first to study the modulation of the medial anterior prefrontal response to pain by opioid administration. From 11 healthy male subjects, we observed an attenuation of the decrease in HbO signal (as assessed by fNIRS) over the medial BA 10 after the administration of oral morphine. The BA 10 signal attenuation was associated with a reduction in the pain-elicited activation in contralateral primary somatosensory cortex (albeit a smaller sample size *N* = 5). Such attenuation of BA 10 response to noxious stimuli was absent after the administration of oral placebo. The results provide further support for a role of BA 10 in pain/nociception.

### Cortical Responses in BA 10 and S1 to Noxious and Innocuous Stimuli

In this study, we evaluated the effects of morphine on the fNIRS-measured signals mainly on two brain regions, i.e., the medial FP cortex (medial BA 10) and the primary somatosensory (S1) cortex. We did not present the results from the left lateral PFC as no significant response to the applied electrical stimuli was observed.

While the role of BA 10 in pain perception has not been fully elucidated, our prior data using fNIRS on awake healthy subjects, patients under light sedation, and patients under general anesthesia showed changes in BA 10 to external painful events that seemed to be consistent across the different levels of consciousness (Yücel et al., 2015; Becerra et al., 2016; Kussman et al., 2016). Support for the involvement of BA 10 in pain includes the following: (a) direct anatomical connections exist between the frontal lobe and other brain regions involved in nociceptive processing, including thalamus and posterior insula which process sensory-discriminative information (Petrides and Pandya, 2007; Burman et al., 2011) and ACC which has been linked to affective-motivational processing (Petrides and Pandya, 2007; Etkin et al., 2011; Orr et al., 2015); (b) the FP cortex sends afferent projections to brain regions (such as the periaqueductal gray, PAG) within the antinociceptive brain network (An et al., 1998; Hadjipavlou et al., 2006), potentially modulating (inhibiting under normal conditions) nociceptive activation of neurons in the dorsal horn of the spinal cord; (c) numerous functional imaging studies have reported robust levels of activations or deactivations in BA 10 and/or adjacent areas across various pain models under both acute and chronic conditions (e.g., Becerra et al., 1999; Tracey et al., 2000; Apkarian et al., 2001; Derbyshire et al., 2002; Lui et al., 2008; Loggia et al., 2012; Peyron et al., 2013; Yoon et al., 2014), see our recent review (Peng et al., 2018a)) and (d) while a breakdown of brain functional connectivity has been reported during anesthesia for most systems, this is not the case for sensory connectivity (Bonhomme et al., 2016). Taken together, although not well defined, the evidence suggests BA 10 is involved in pain/nociceptive processing either through direct or indirect pathways.

The S1 is a classic sensory region known to be involved in the nociceptive pathway and responsive to opioid administration (see below). For S1, previous studies have observed activation of this region following experimental painful/nociceptive stimuli (Becerra et al., 2001; DaSilva et al., 2002; Jantsch et al., 2005; Moulton et al., 2005).

### Effects of Morphine on BA 10 and S1 Responses to Noxious and Innocuous Stimuli

The primary focus of this study was to evaluate whether a well-known analgesic, morphine, would affect the fNIRS-measured response to pain in the brain regions that we had previously characterized in healthy subjects and patients (i.e., the medial BA 10). Here we focus on the effects of blockade of nociceptive stimuli with oral morphine. Due to placebo effects, the study design was double blind and randomized. Notably, subjects served as their own control. Although drugs may have an unknown prolonged effect on neural systems, we assumed that at least a two-week washout interval between drug and placebo administration would be sufficient given the short half-life of morphine (i.e., within hours).

Morphine is an opioid analgesic that is considered as a gold standard analgesic for nociceptive stimuli (Stein et al., 2000; Ruiz-Garcia and Lopez-Briz, 2008; Dietis et al., 2009). It acts on μ-receptors that are located in both the peripheral and central nervous system (CNS). Within the CNS, morphine is observed to inhibit nociceptive stimuli at the spinal cord, brainstem (e.g., PAG), subcortical (e.g., thalamus) and several cortical areas (Yaksh, 1981; Lipp, 1991; Al-Hasani and Bruchas, 2011). For example, in preclinical studies using electrophysiological measures of neural activation produced by painful stimuli, morphine was reported to inhibit neuronal activity to pain evoked in a number of these regions including the S1, thalamus (the ventral posterolateral nucleus and the mediodorsal nucleus), and ACC (Wang et al., 2009). In human imaging studies, inhibitive effects of morphine on the pain-induced cortical activations were previously seen in the ACC, the insula and the inferior parietal cortex (Becerra et al., 2006; Hansen et al., 2015). To date, little data is available on the modulation of morphine on brain deactivations or on BA 10 response associated with pain. In this study, morphine was observed to attenuate the BA 10 deactivation to noxious stimuli in the medial BA 10, but had minimal effects to innocuous stimuli. We specifically repeated fNIRS data collection over a 90 min post-administration period as the time taken to reach the maximum plasma concentration (T_max_) for immediate-release morphine has been reported to be around 1 h with a range of 20–30 min from its administration (Collins et al., 1998). As noted, the maximal effect of medial BA 10 signal attenuation (and also for signal in the contralateral S1) was observed to be within this time range.

An understanding of the effects of morphine on BA 10, as alluded to above, is more complex and less well understood. We believe that the observed signal attenuation is primarily associated with inhibition of the afferent nociceptive signal. However, the contribution of BA 10 to the conscious response of the signal may involve a number of complex processes, as discussed below. First, the deactivation of BA 10 following noxious stimuli has been attributed to the inhibition of the DMN, which is potentially associated with the reorientation of a subject’s attention to the salience of the external painful event (Kucyi et al., 2013). The deactivation of the DMN has been reported to be under inhibitive control of the salience network (SN; Chen et al., 2013; Wen et al., 2013). When morphine is administered, the diminished pain sensation may reduce the level of activation of the SN, which potentially leads to an attenuation of the DMN deactivation. Second, opioids such as morphine are known to impair cognitive functions (Strand et al., 2017). This may be carried out by altering the fronto-parietal and medio-lateral connectivity of the brain cognitive networks (Hudetz, 2012; Khalili-Mahani et al., 2012), including the DMN which has been seen to be important in the maintenance of a subject’s cognitive performance (Eichele et al., 2008; Prado and Weissman, 2011; Lin et al., 2015). The reduced level of medial BA 10 deactivation observed in this study may reflect the disruption of the DMN and the impairment of cognitive functions as a result of morphine administration. Third, BA 10 is known to have connections with many above-mentioned brain regions involved in opioid-induced analgesia. For instance, opioids in the ACC, a rostral frontal region that shares extensive connections with BA 10, have been shown to play an important role in nociceptive pain in animals (Navratilova et al., 2015) and humans (Petrovic et al., 2002). While the medial BA 10 and the ACC have been revealed to be critical in the regulation of the affective-motivational dimension of pain (Coghill et al., 2003; Bushnell et al., 2013), Price et al. (1985) reported that morphine, even administered at a low dose (e.g., 0.04 mg/kg intravenous administration), is able to significantly reduce the affective component of pain, which may therefore potentially lead to decreased BA 10 activity related to emotion regulations. Finally, opioid antagonists (e.g., naloxone) seemed to produce opposite changes in the prefrontal regions related to endogenous pain modulation (e.g., lateral BA 10 and BA9) when compared with morphine (Taylor et al., 2013). This implies that morphine may modulate the endogenous pain modulation system and alter the BA 10 response through interactions within the prefrontal areas. Taken together, the results of this study provide further evidence to support the notion that BA 10 may play an integrative role and be involved in the high-level processing of pain (Peng et al., 2018a).

For S1, it should be noted that this region has a low density of μ-opioid receptors and therefore has less binding potential for acute administration of the short acting μ-opioid agonists (e.g., remifentanil as reported in Leppä et al. (2006)). However, the modulation of its response by opioids may take place through thalamo-cortical inhibition or activation of the descending modulation, which inhibits its afferent nociceptive traffic. Although not consistently observed, the suppression of S1 activity after morphine administration has been reported previously (Wang et al., 2009). Notably, in another study, the opioid alfentanil (a short acting μ-opioid) produced a graded decrease in S1 activation in response to pain with increasing opioid concentrations (Oertel et al., 2008). With its analgesic response better understood, the diminished S1 activations, which were in parallel with the observed BA 10 response (albeit a smaller sample size, i.e., 5 compared with 11 in BA 10 analysis), may serve as a “control” for the expected analgesic effects of morphine on the brain.

### Pain Rating and BA 10 Response

Five randomly selected subjects were asked to report subjective scores of pain perception level after each scan of morphine or placebo visit. Our initial intent through this study was to determine if subjects’ conscious scoring would make a difference to the fNIRS-measured brain signal. From the pain scores of the five subjects, we observed a larger decrease in the VAS ratings of the perceived pain following the administration of morphine than placebo (Supplementary Figure S1). However, the difference between the two visits did not reach statistical significance (*p* > 0.05 in one-tailed paired *t*-test). One explanation is that the limited sample size may make the significance level of the test more susceptible to a few outlier ratings (see limitations below). Moreover, although prior studies have validated the use of pain intensity rating scales with regards to the responsivity (Ferreira-Valente et al., 2011), the VAS rating of intensity is only one measure of pain perception. The evaluation of brain response to pain, on the other hand, may confer the more complex nature of the experience. For example, previous work using fMRI to study the effect of naloxone on CNS activity reported significant changes in the CNS response to noxious heat after the administration of naloxone compared to placebo, but with no statistical difference in the VAS reports of perceived pain intensity between conditions (Borras et al., 2004). Given that the medial PFC (BA 10) represents integrative information about pain stimulus (Peng et al., 2018a), it is possible that the observed attenuation of BA 10 deactivation following pain in this study is more associated with nonsensory emotional or attentional processing of pain in the brain (see section “Effects of Morphine on BA 10 and S1 Responses to Noxious and Innocuous Stimuli” above). Despite the low number of subjects and the lack of statistical significance in VAS ratings, we believe that the observed attenuation of BA 10 signal following morphine administration was a part of the analgesic response of the brain due to the following reasons: (a) the temporal profile of the attenuation of the BA 10 response to noxious stimuli closely matched the pharmacokinetic-pharmacodynamic model of oral morphine, which has been studied in the literature and has been well established (Collins et al., 1998; Staahl et al., 2008); (b) although no statistical significance was obtained, a clear graded decrease in the averaged VAS pain ratings of the experienced painful events was observed in the morphine visits, reaching maximum reduction at 60 min after drug administration. This decrease also matched the pharmacokinetic-pharmacodynamic model of oral morphine and could not be observed after placebo was given (Supplementary Figure S1) and (c) in the small comparison between the five subjects who were asked to rate his pain and five other subjects who were not, we did not observe major difference in the brain response modulated by morphine or placebo (Supplementary Figure S15).

### Limitations

There are a number of limitations related to this study. These include:

a. *Pain habituation*: pain habituation describes a phenomenon where pain and its associated response show a progressive reduction over continuous or repetitive stimulation trials (Bingel et al., 2007; Greffrath et al., 2007; Rennefeld et al., 2010). It has been considered as a self-protection mechanism against the development of chronic pain states (Rennefeld et al., 2010). In this study, each subject received a total number of 24 painful stimuli in four fNIRS scans during one single visit (morphine or placebo). The habituation to the stimuli may produce a gradual reduction in the pain-induced brain response, which then leads to an attenuation of the fNIRS signal. However, we believe that pain habituation should not be a major factor that drove the reported fNIRS-measured brain signal change following morphine administration. First, we observed a recovery of the pain response in both the medial BA 10 and the right S1 in the 90 min post-scan. The U-shaped temporal profile of the brain pain response was not in line with the hypothesis of pain habituation (which should exhibit a “steady decrease in response magnitude” (Greffrath et al., 2007)) but closely follow the pharmacodynamic curve of morphine. Moreover, our previous study with a similar experimental design has suggested that a relatively long resting period between scans (e.g., 30 min) might be able to eliminate the effect of habituation in some subjects (Peng et al., 2018b).
b. *Confounding factors in opioid analgesia*: opioid is known to potentially depress respiration and lead to hypercapnia (elevated carbon dioxide concentration in the blood; Pattinson et al., 2009). Furthermore, the baseline cortical hemodynamics such as cerebral blood flow (CBF) and cerebral blood volume (CBV) levels may also be altered by certain analgesics (e.g., remifentanil; Wagner et al., 2001; Lorenz et al., 2002; Macintosh et al., 2008). These confounding factors were not modeled in this particular study. Instead, we employed a short separation approach by which we regressed out the extracerebral signals to mitigate the effect of any global changes in resting state or evoked hemodynamics (such as a global increase in blood CO_2_ levels following the administration of an opioid). Future work explicitly controlling for subjects’ cardiorespiratory parameters and CBF/CBV levels may be beneficial to more precisely delineate the effect of morphine on the BA 10 pain signal.
c. *Sample size*: this study was conducted in a relatively small and restricted group of subjects (all males, *N* = 11 for BA 10 and *N* = 5 for S1). Moreover, we did not perform motion correction on most of the data for the purpose of reducing signal magnitude changes induced during pre-processing. Instead, stimulation trials that presumably overlapped with artifacts were simply excluded from the analysis. The limited sample size and number of stimuli included in each scan may lower the statistical significance and increase the risk of generating false positive results (especially for S1). The fixed effect model used in the GLM group analysis led to higher statistical scores in the group-level activation maps that allowed clear differentiation of the brain pain response among the four scans before and after morphine administration. However, neglecting the inter-subject variability and applying a fixed effect model (rather than using a random effect model) limited the ability of extending our GLM findings to a more generalized subject group. Studies including a larger sample size and both genders of subjects (Sarton et al., 2000) may be necessary to further validate the results of this work.

## Conclusion

The deactivation in the medial FP cortex (medial BA 10) associated with pain has been observed in previous studies but has not been well understood. In this study, we observed that such medial BA 10 response to pain could be modulated by the administration of oral morphine. The attenuation of the signal closely matched the expected temporal profile of the blood plasma concentration for the drug. These results provide further evidence in supporting the role of medial BA 10 in the process of pain perception, whether it be sensory, affective or attentional (see Peng et al., 2018a). Our results also reveal the potential of using fNIRS to objectively evaluate pain and analgesic efficacy.

## Author Contributions

DB, LB and DAB conceptualized this study. KP, MY, CA, DB, LB and DAB designed the methodology for the study. KP, MY, SS, EB, CA, MH, AL and EG participated in the data acquisition and data curation. KP, MY, DB, LB and DAB analyzed and interpreted the data. KP and DB drafted the manuscript. MY, CA, LB, MH, AL and DAB critically revised the manuscript for content.

## Conflict of Interest Statement

DAB is an inventor of the technology licensed to TechEn, Inc., which is a company that provides solutions to noninvasive optical brain imaging. DAB’s interests were reviewed and are managed by Massachusetts General Hospital and Partners HealthCare in accordance with their conflict of interest policies. The remaining authors declare that the research was conducted in the absence of any commercial or financial relationships that could be construed as a potential conflict of interest.
