# Supplementary material for: Morphine Attenuates fNIRS Signal Associated With Painful Stimuli in the Medial Frontopolar Cortex (medial BA 10)
Source: Front Hum Neurosci. 2018 Oct 4;12:394. doi: 10.3389/fnhum.2018.00394 (PMC6186992; doi:10.3389/fnhum.2018.00394)
Supplement: Supplementary file 1 [file Data_Sheet_1.docx]

Supplementary Material

Morphine Attenuates fNIRS Signal Associated with Painful Stimuli in the Medial Frontopolar Cortex (medial BA 10)

Ke Peng*, Meryem A. Yücel, Sarah C. Steele, Edward A. Bittner, Christopher M. Aasted, Mark A. Hoeft, Arielle Lee, Edward E. George, David A. Boas, Lino Becerra, David Borsook

*** Correspondence:** Ke Peng: Ke.Peng@childrens.harvard.edu

# Supplementary Figures and Tables

## Supplementary Figures

**
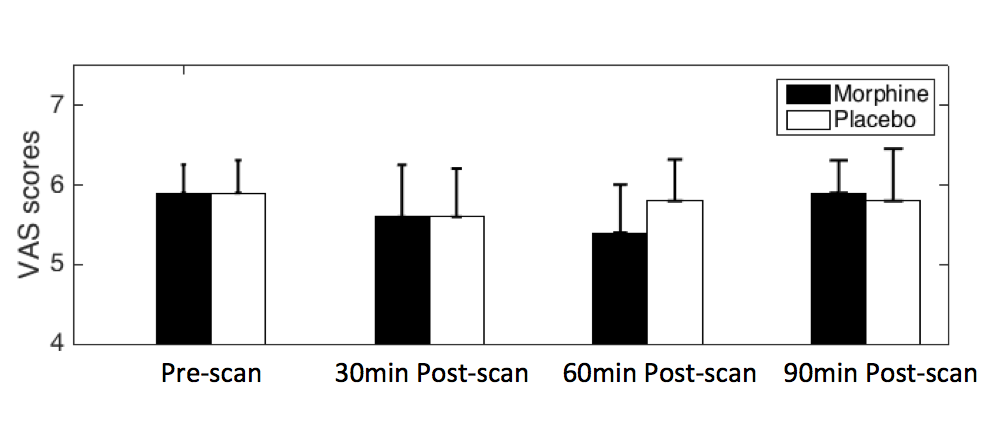
**

**Supplementary Figure 1.** VAS ratings of perceived pain levels during the morphine visits and the placebo visits. Error bars indicate the standard errors of the mean. These scores were collected separately from 5 randomly selected subjects.

**
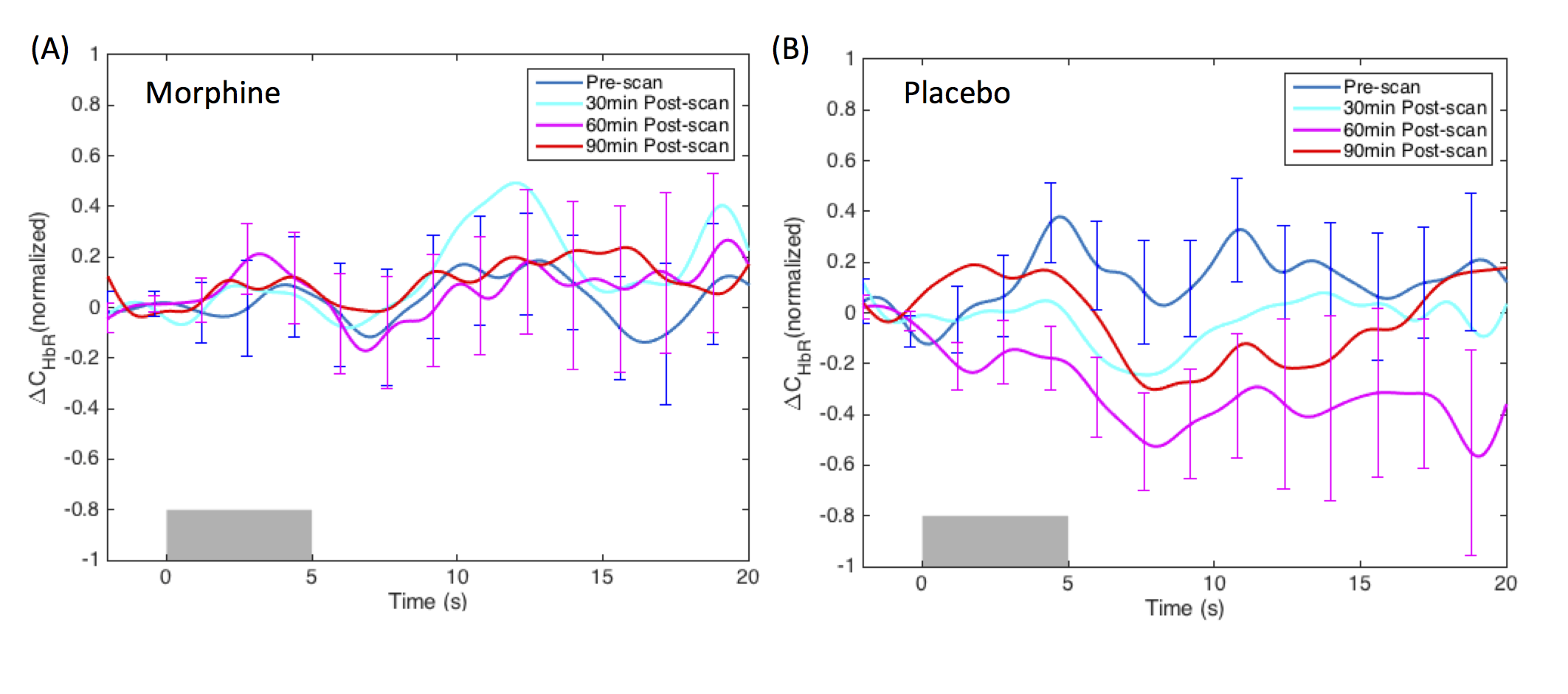
**

**Supplementary Figure 2.** Normalized HbR response to noxious stimuli (VAS7) in the medial BA 10 during morphine visits (A) and placebo visits (B). Gray bars indicate the time period when noxious electrical stimuli were applied. All error bars show the standard error of the mean.


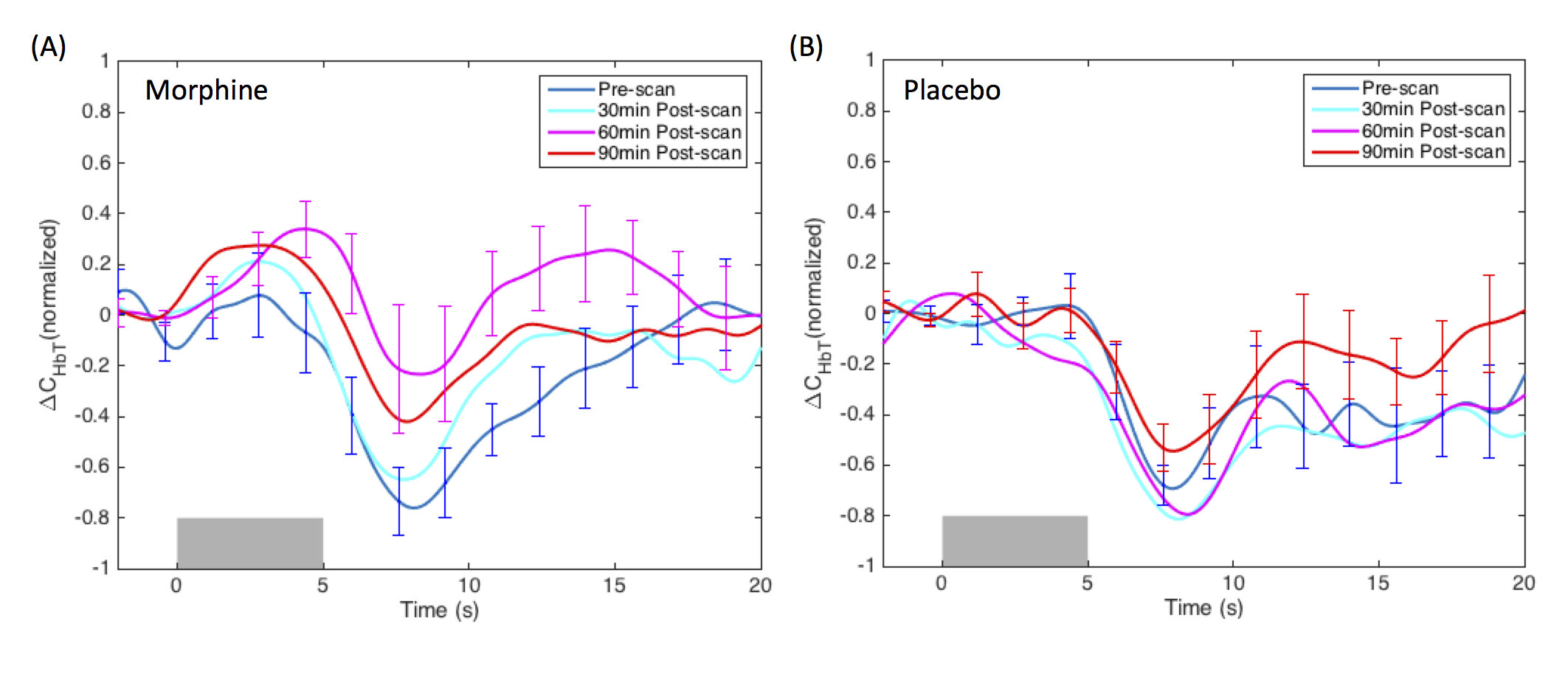


**Supplementary Figure 3.** Normalized HbT response to noxious stimuli (VAS7) in the medial BA 10 during morphine visits (A) and placebo visits (B). Gray bars indicate the time period when noxious electrical stimuli were applied. All error bars show the standard error of the mean.


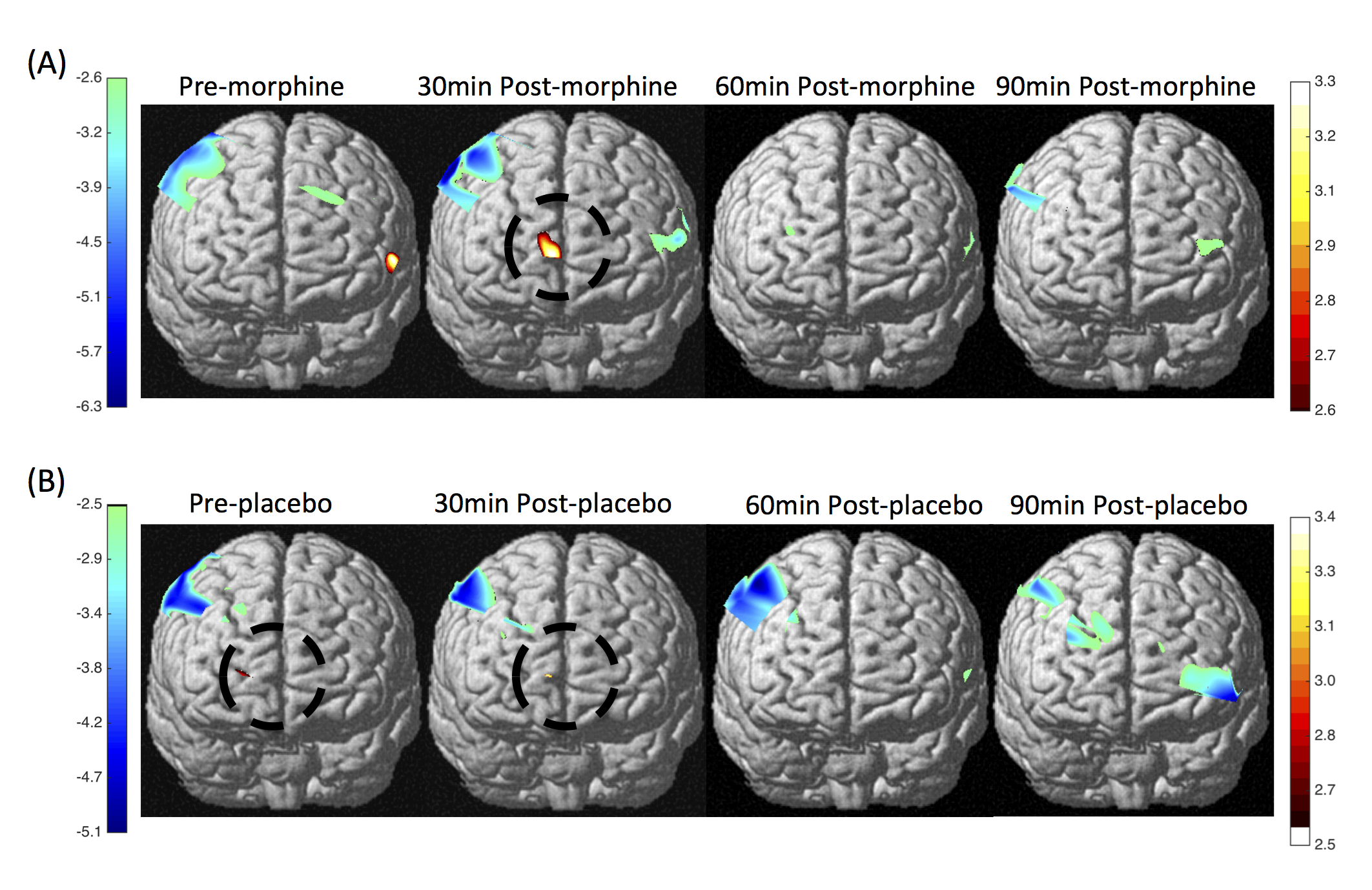


**Supplementary Figure 4.** HbR contrast maps in the medial BA 10 associated with noxious stimuli (VAS7) generated from the GLM analysis. (A) Group-level statistical parametric maps during the visits when the subjects received oral morphine; (B) Group-level statistical parametric maps during the visits when the subjects received oral placebo, right view, pFDR corrected, p<0.05. Identified HbR activation clusters are circled in black.


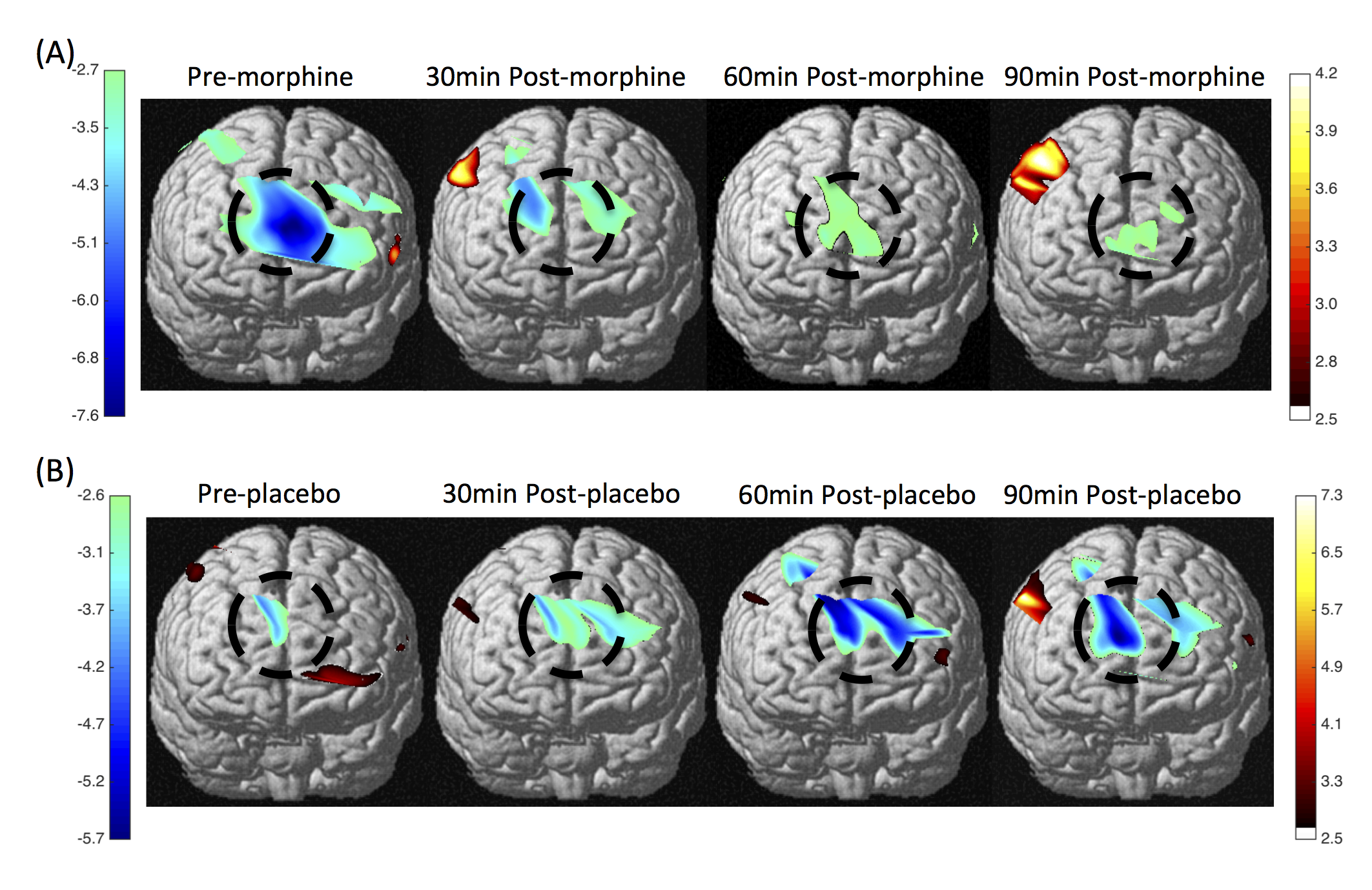


**Supplementary Figure 5.** HbT contrast maps in the medial BA 10 associated with noxious stimuli (VAS7) generated from the GLM analysis. (A) Group-level statistical parametric maps during the visits when the subjects received oral morphine; (B) Group-level statistical parametric maps during the visits when the subjects received oral placebo, right view, pFDR corrected, p<0.05. Identified HbT deactivation clusters are circled in black.


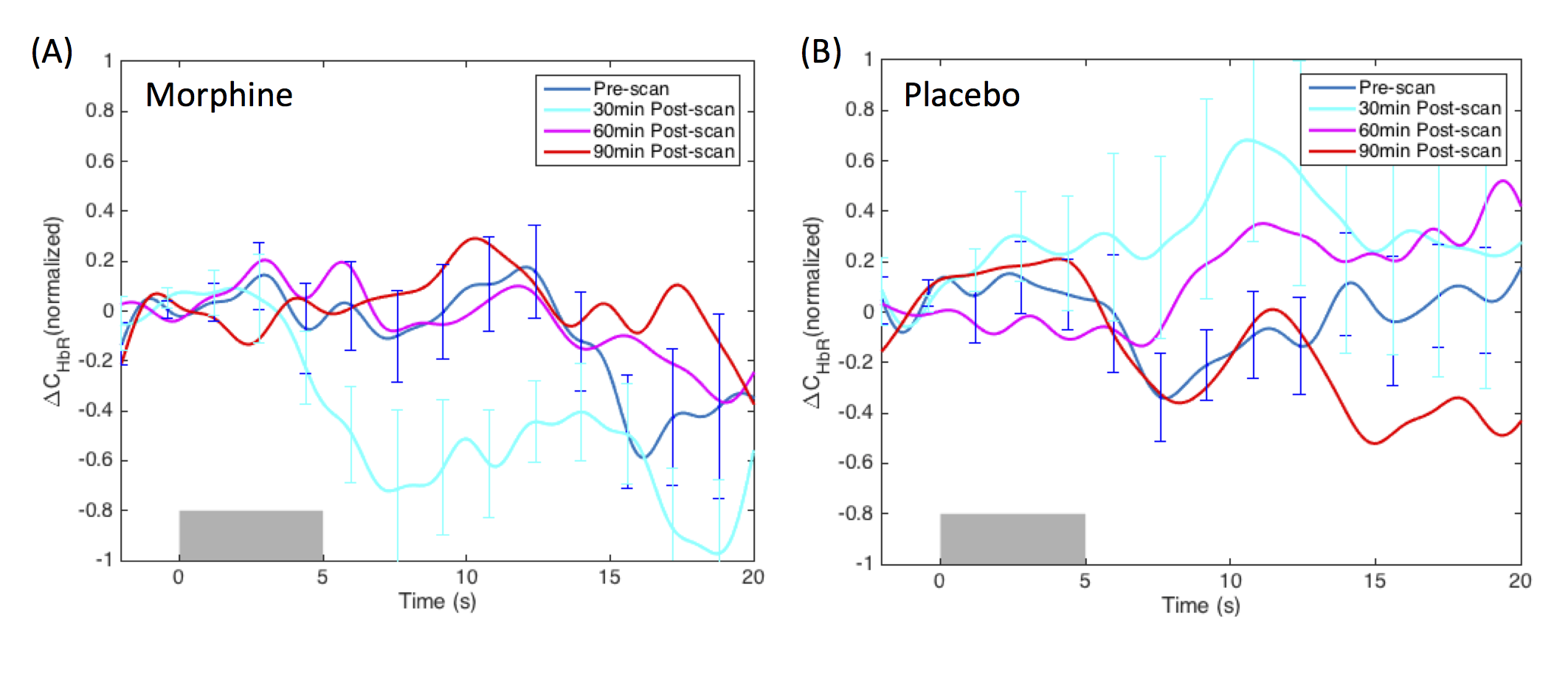


**Supplementary Figure 6.** Normalized HbR response to innocuous stimuli (VAS3) in the medial BA 10 during morphine visits (A) and placebo visits (B). Gray bars indicate the time period when innocuous electrical stimuli were applied. All error bars show the standard error of the mean.


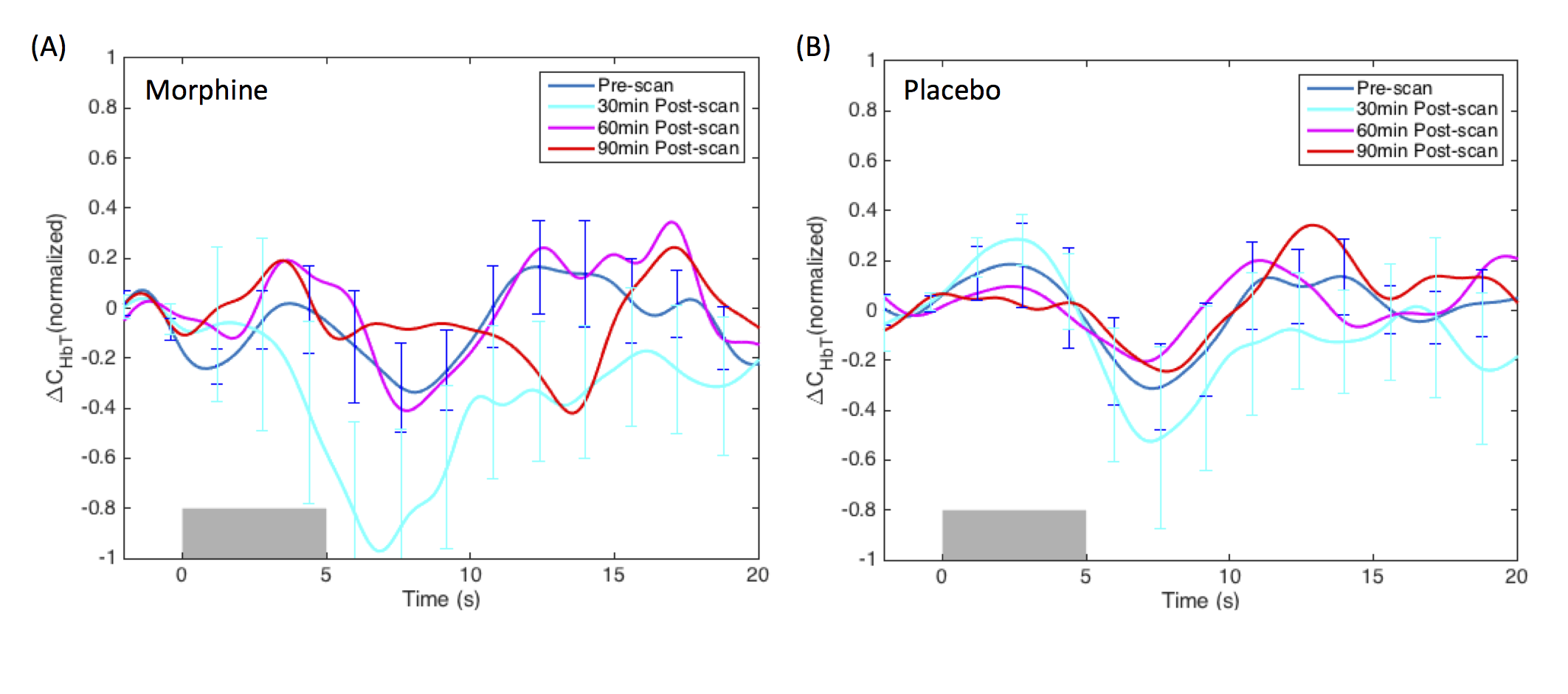


**Supplementary Figure 7.** Normalized HbT response to innocuous stimuli (VAS3) in the medial BA 10 during morphine visits (A) and placebo visits (B). Gray bars indicate the time period when innocuous electrical stimuli were applied. All error bars show the standard error of the mean.


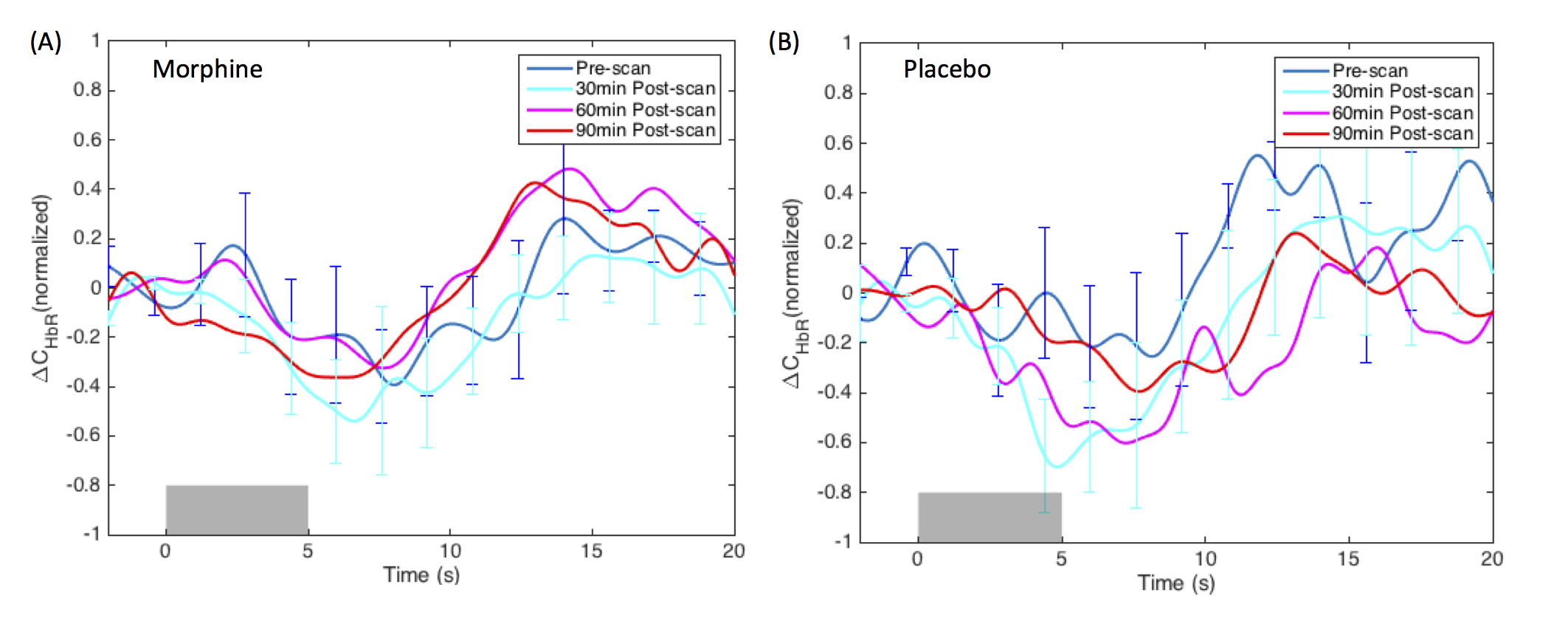


**Supplementary Figure 8.** Normalized HbR response to noxious stimuli (VAS7) in the right S1 during morphine visits (A) and placebo visits (B). Gray bars indicate the time period when noxious electrical stimuli were applied. All error bars show the standard error of the mean.


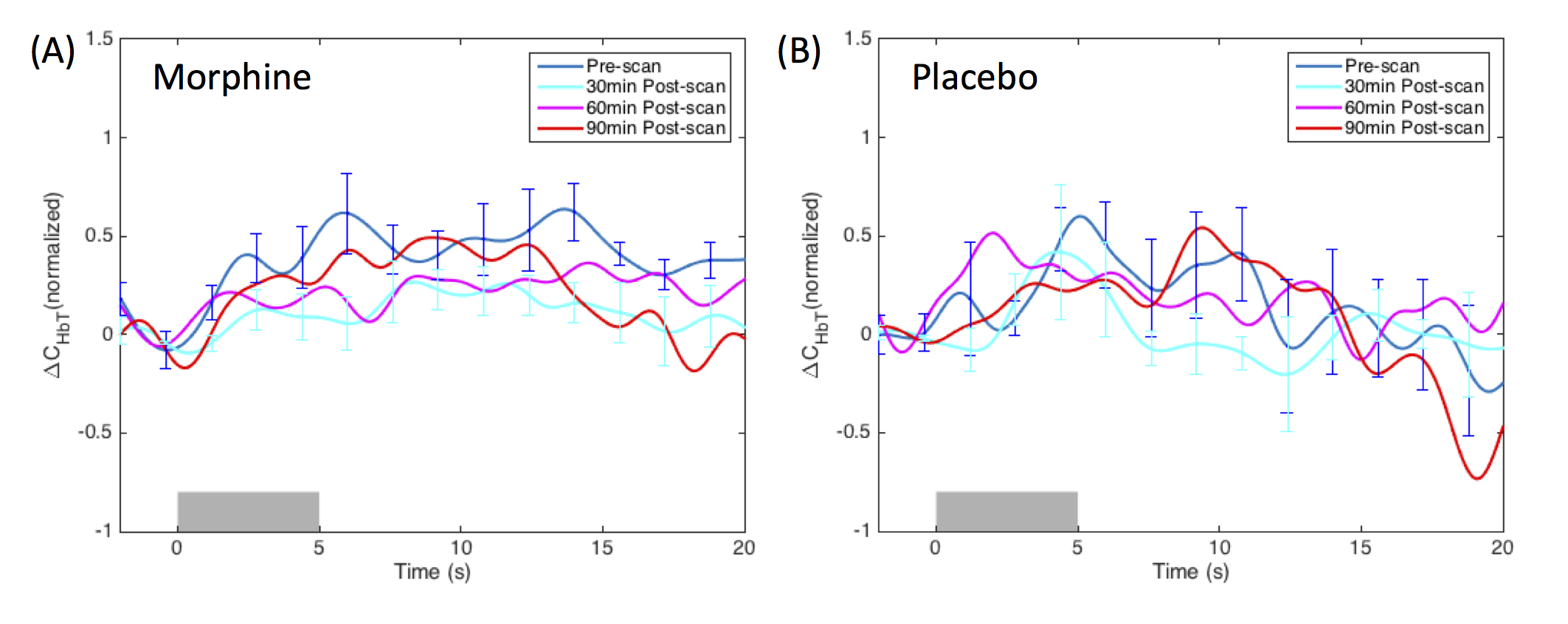


**Supplementary Figure 9.** Normalized HbT response to noxious stimuli (VAS7) in the right S1 during morphine visits (A) and placebo visits (B). Gray bars indicate the time period when noxious electrical stimuli were applied. All error bars show the standard error of the mean.


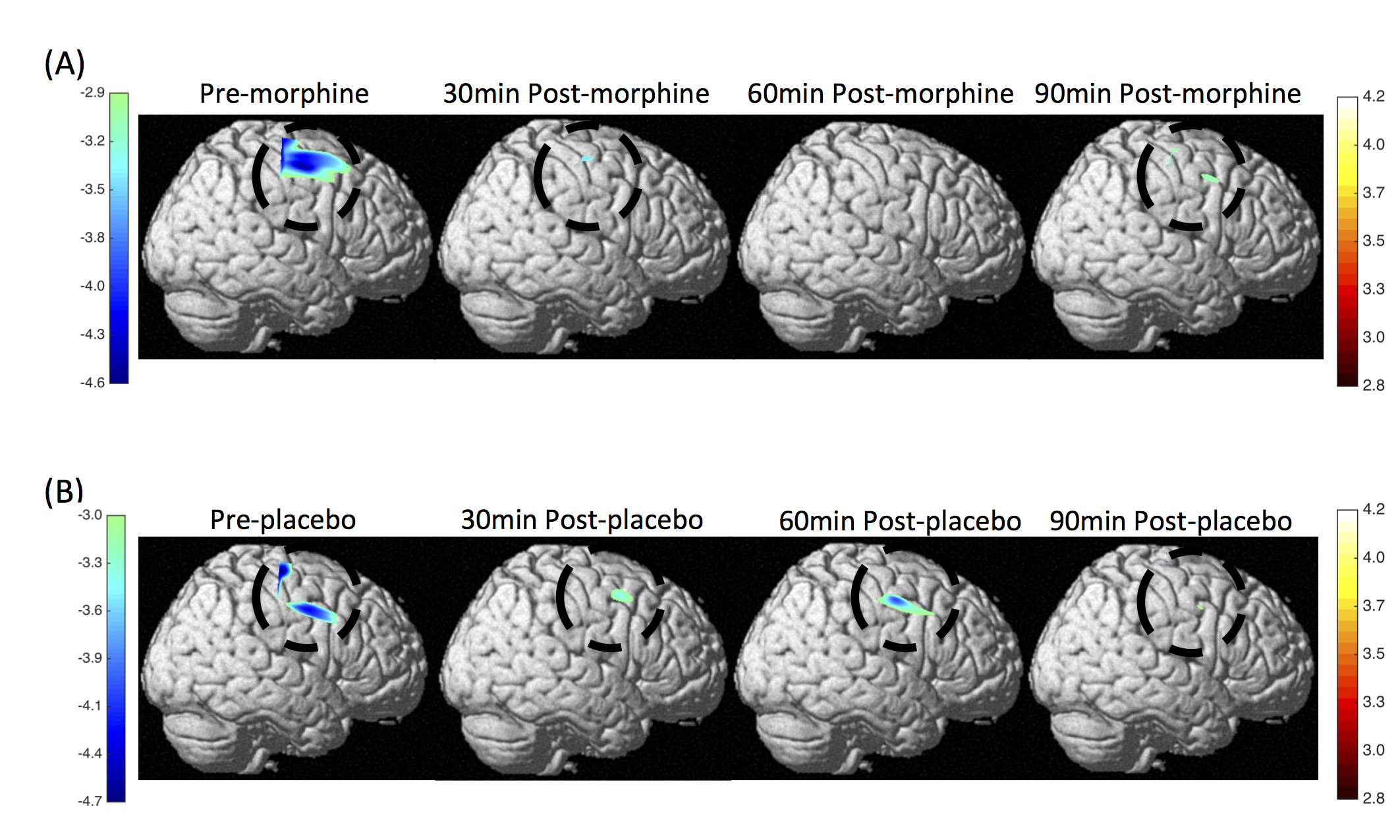


**Supplementary Figure 10.** HbR contrast maps in the right S1 associated with noxious stimuli (VAS7) generated from the GLM analysis. (A) Group-level t-statistical maps during the visits when the subjects received oral morphine; (B) Group-level t-statistical maps during the visits when the subjects received oral placebo, right view, pFDR corrected, p<0.05. In the cases that pFDR did not find a threshold, a fixed threshold of |t| > 3 was applied. Identified HbR deactivation clusters are circled in black.


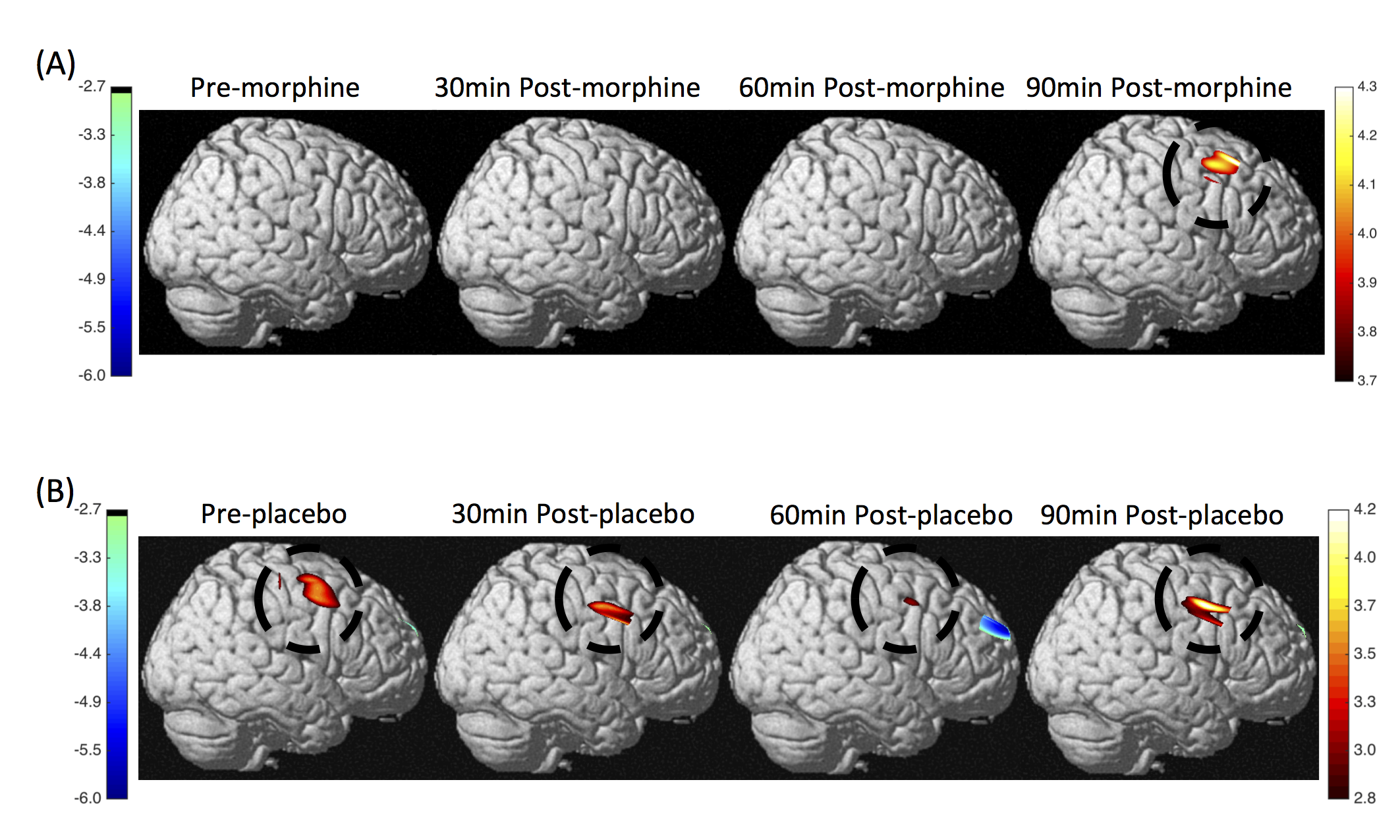


**Supplementary Figure 11.** HbT contrast maps in the right S1 associated with noxious stimuli (VAS7) generated from the GLM analysis. (A) Group-level t-statistical maps during the visits when the subjects received oral morphine; (B) Group-level t-statistical maps during the visits when the subjects received oral placebo, right view, pFDR corrected, p<0.05. In the cases that pFDR did not find a threshold, a fixed threshold of |t| > 3 was applied. Identified HbT activation clusters are circled in black.


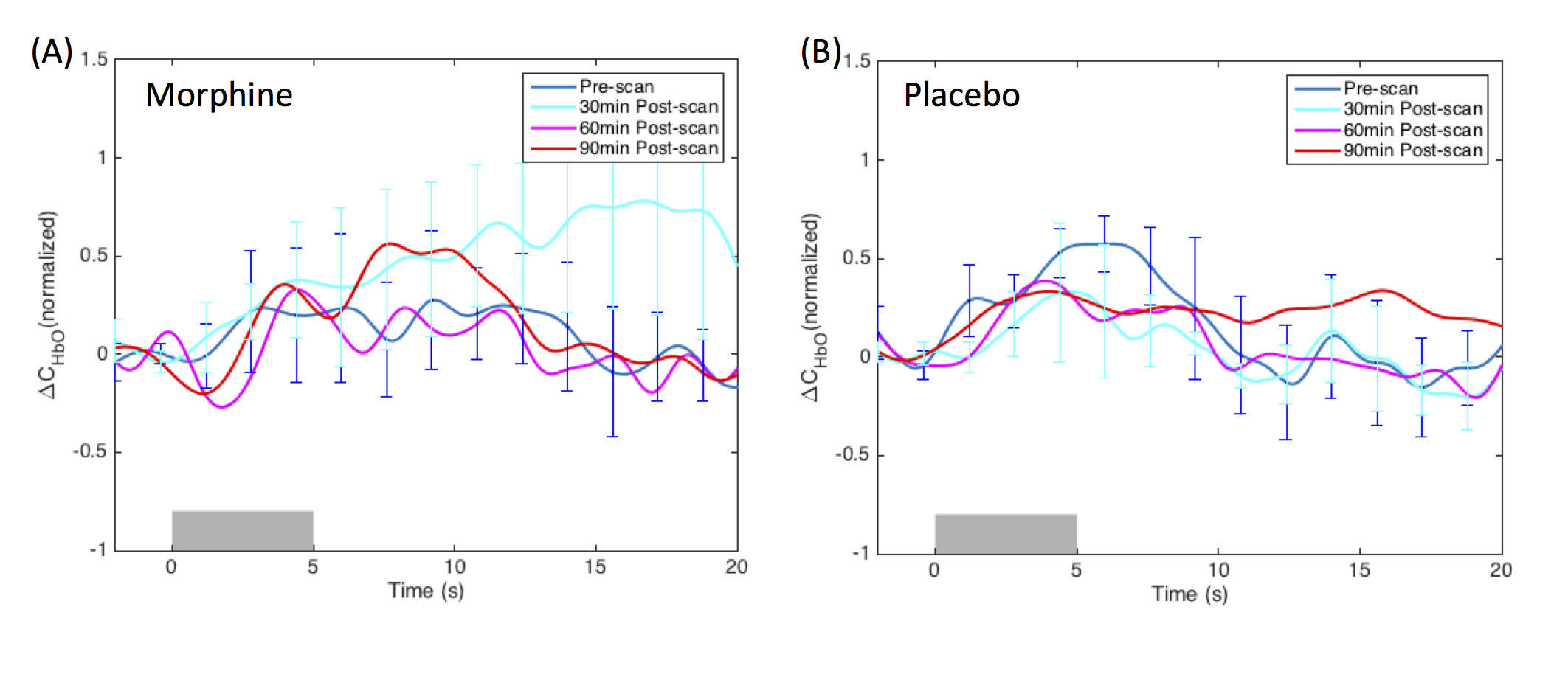


**Supplementary Figure 12.** Normalized HbO response to innocuous stimuli (VAS3) in the right S1 during morphine visits (A) and placebo visits (B). Gray bars indicate the time period when innocuous electrical stimuli were applied. All error bars show the standard error of the mean.


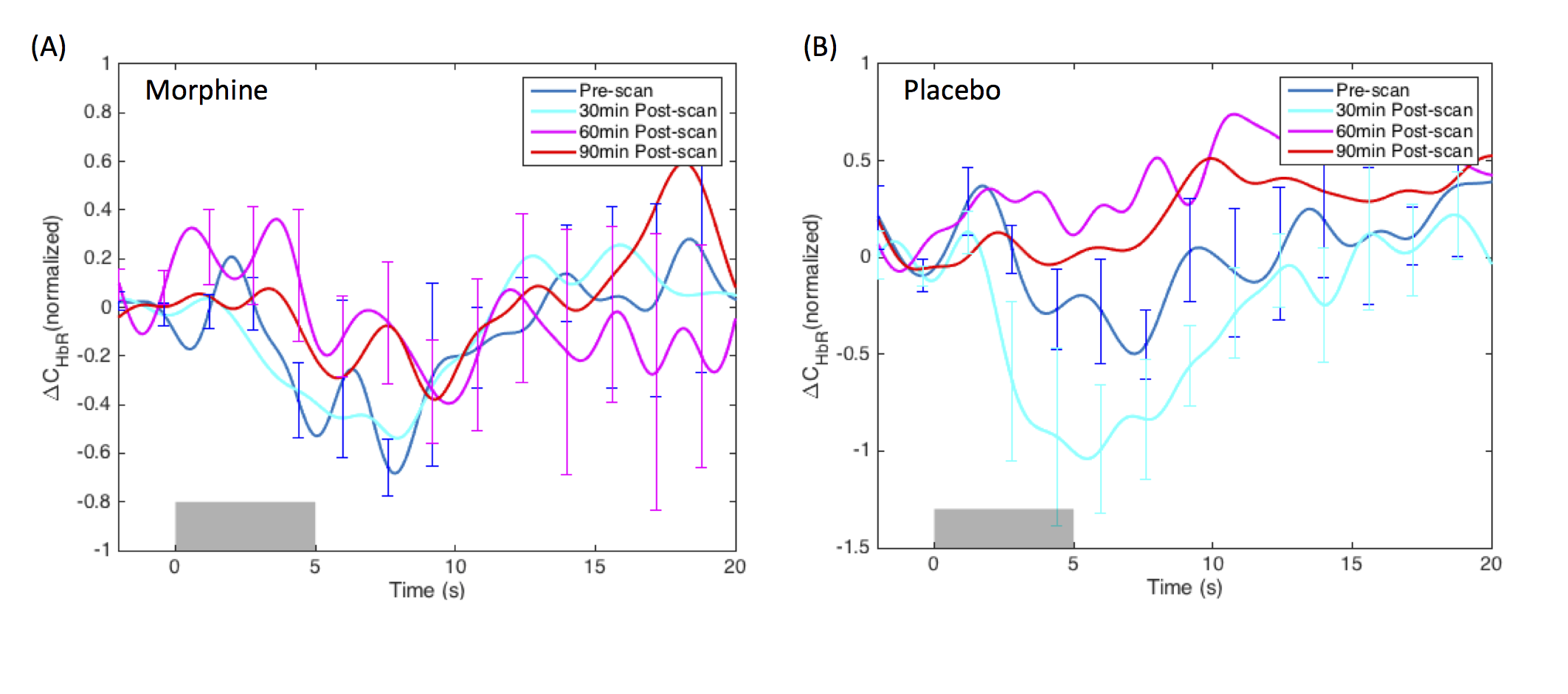


**Supplementary Figure 13.** Normalized HbR response to innocuous stimuli (VAS3) in the right S1 during morphine visits (A) and placebo visits (B). Gray bars indicate the time period when innocuous electrical stimuli were applied. All error bars show the standard error of the mean.


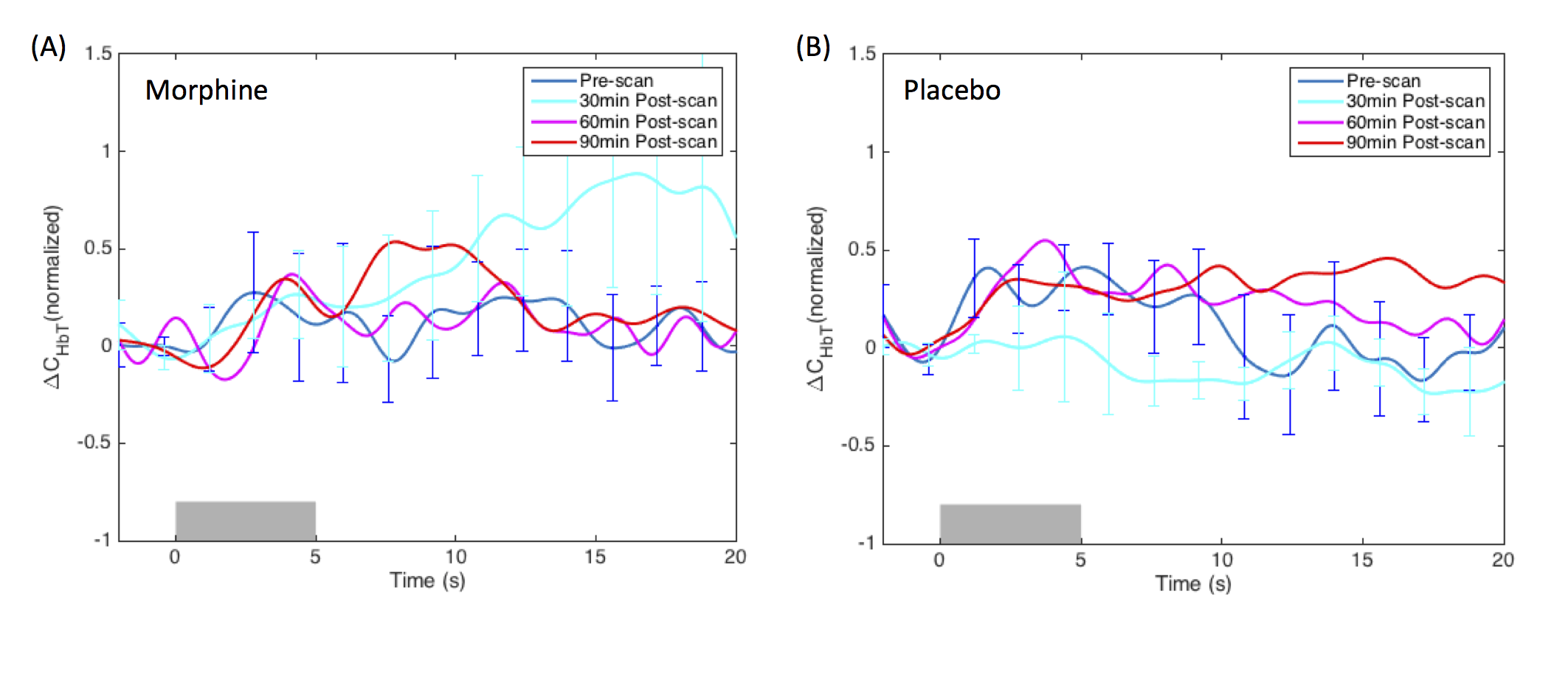


**Supplementary Figure 14.** Normalized HbT response to innocuous stimuli (VAS3) in the right S1 during morphine visits (A) and placebo visits (B). Gray bars indicate the time period when innocuous electrical stimuli were applied. All error bars show the standard error of the mean.


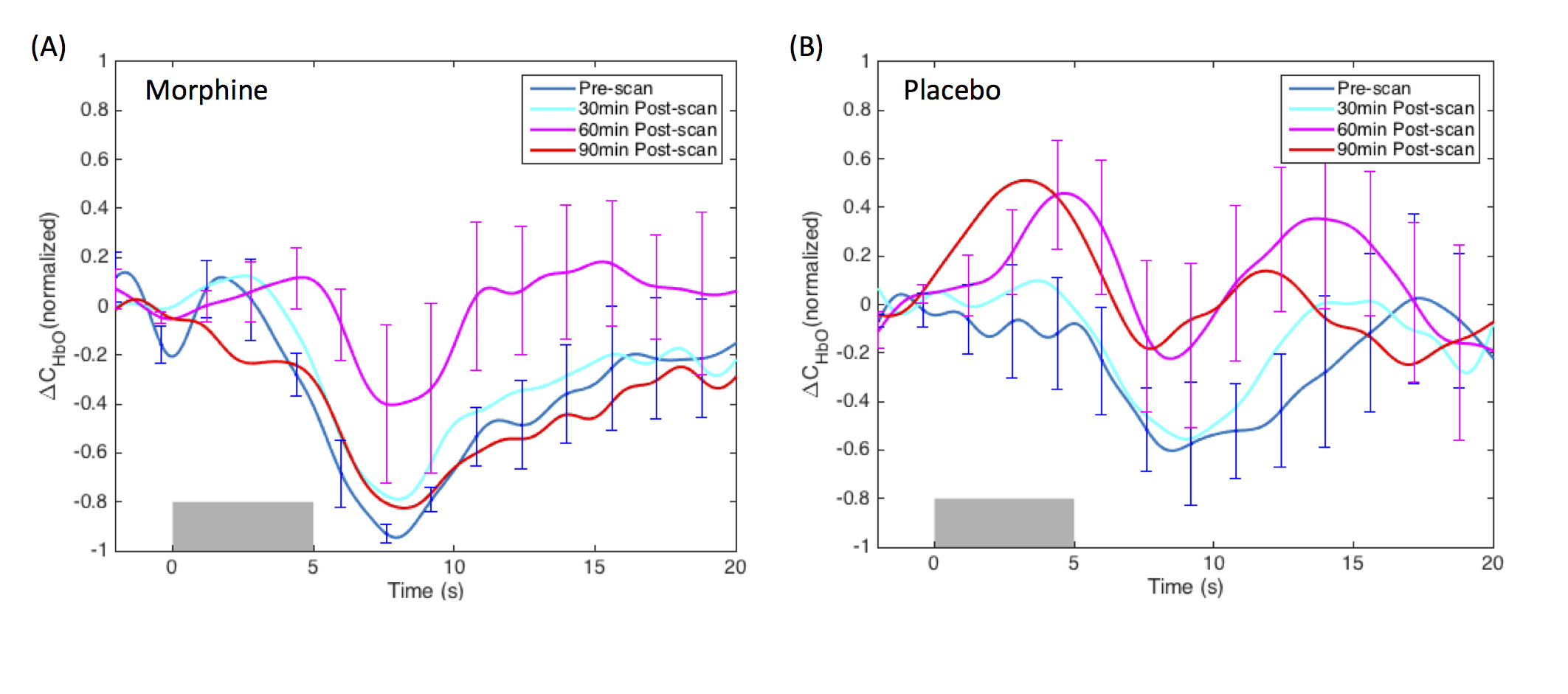


**Supplementary Figure 15.** Normalized HRFs of HbO concentration changes to noxious stimuli (VAS7) obtained from (A) 5 subjects who were asked to rate their level of pain after each scan and (B) from another 5 subjects who were not asked to report pain ratings (one outlier subject has been excluded). Gray bars indicate the time period when noxious electrical stimuli were applied. All error bars show the standard error of the mean. No major difference of morphine effect in terms of the attenuation of BA 10 response to noxious stimuli was observed between the two groups of subjects.


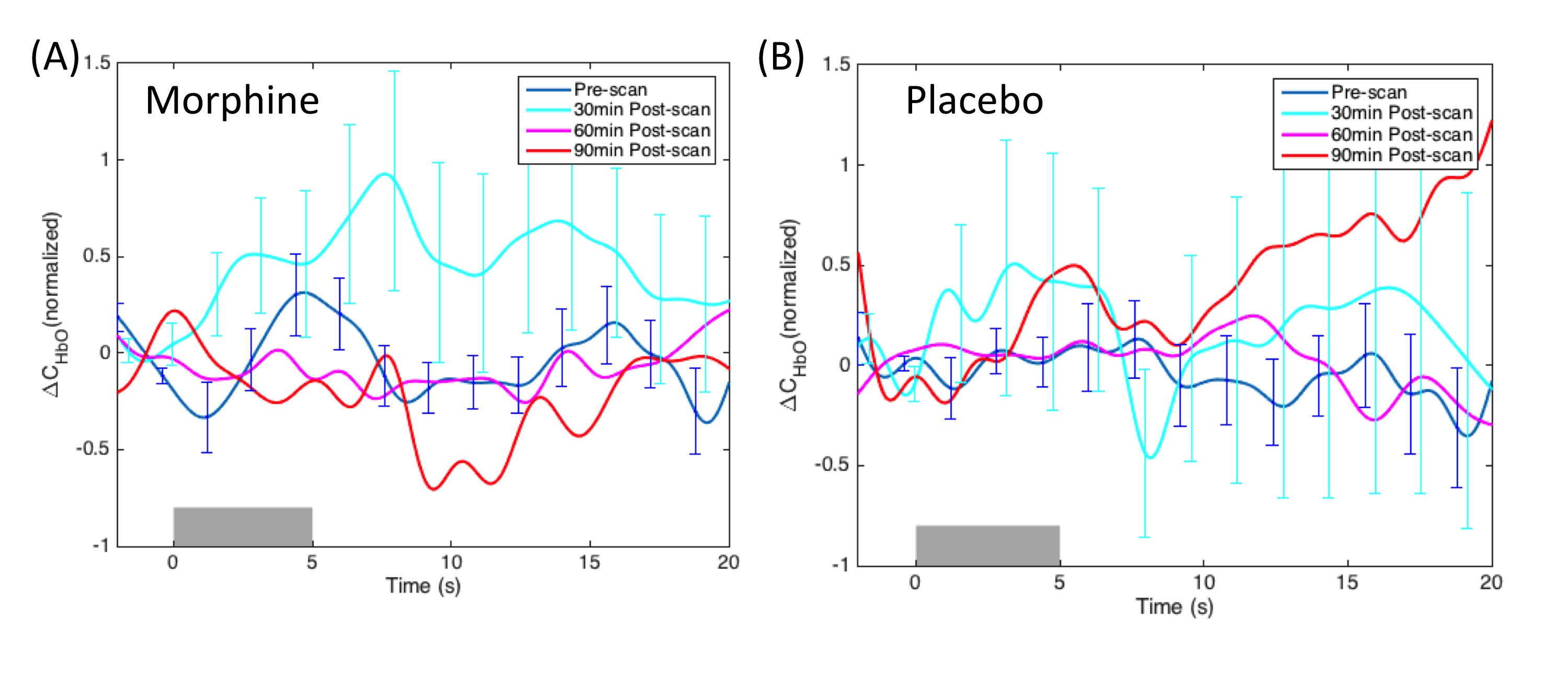


**Supplementary Figure 16.** Normalized HbO response to noxious stimuli (VAS7) in the left lateral prefrontal cortex (averaged from channels 1-8) during morphine visits (A) and placebo visits (B). Gray bars indicate the time period when innocuous electrical stimuli were applied. All error bars show the standard error of the mean.


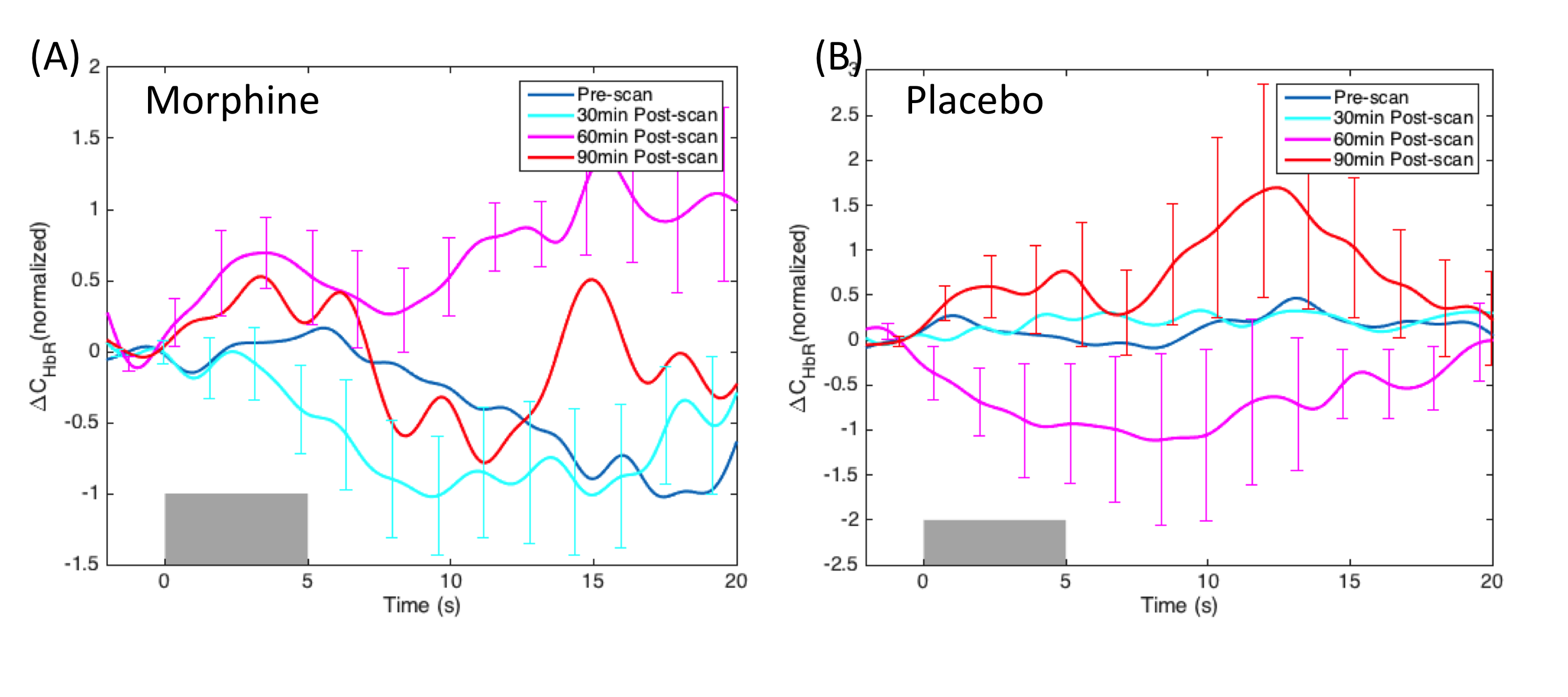


**Supplementary Figure 17.** Normalized HbR response to noxious stimuli (VAS7) in the left lateral prefrontal cortex (averaged from channels 1-8) during morphine visits (A) and placebo visits (B). Gray bars indicate the time period when innocuous electrical stimuli were applied. All error bars show the standard error of the mean.


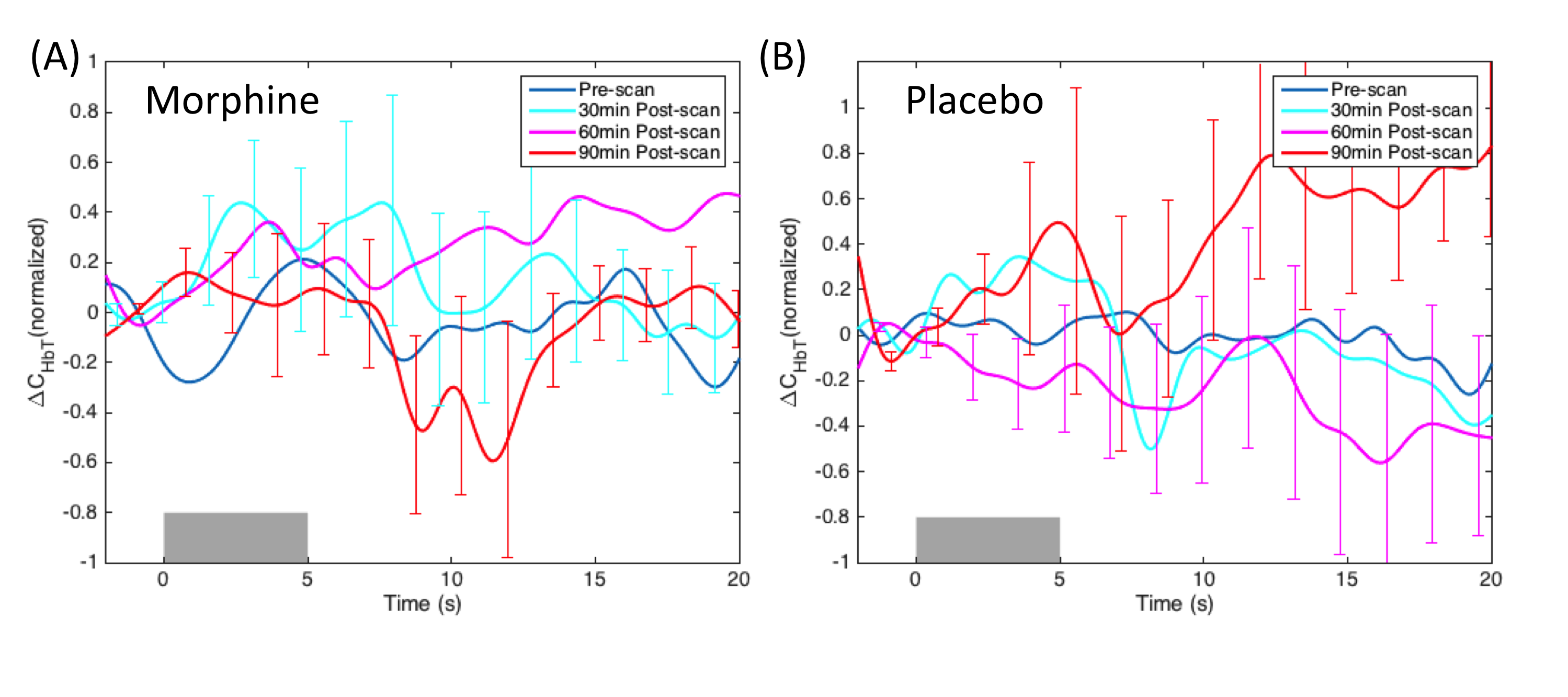


**Supplementary Figure 18.** Normalized HbT response to noxious stimuli (VAS7) in the left lateral prefrontal cortex (averaged from channels 1-8) during morphine visits (A) and placebo visits (B). Gray bars indicate the time period when innocuous electrical stimuli were applied. All error bars show the standard error of the mean.


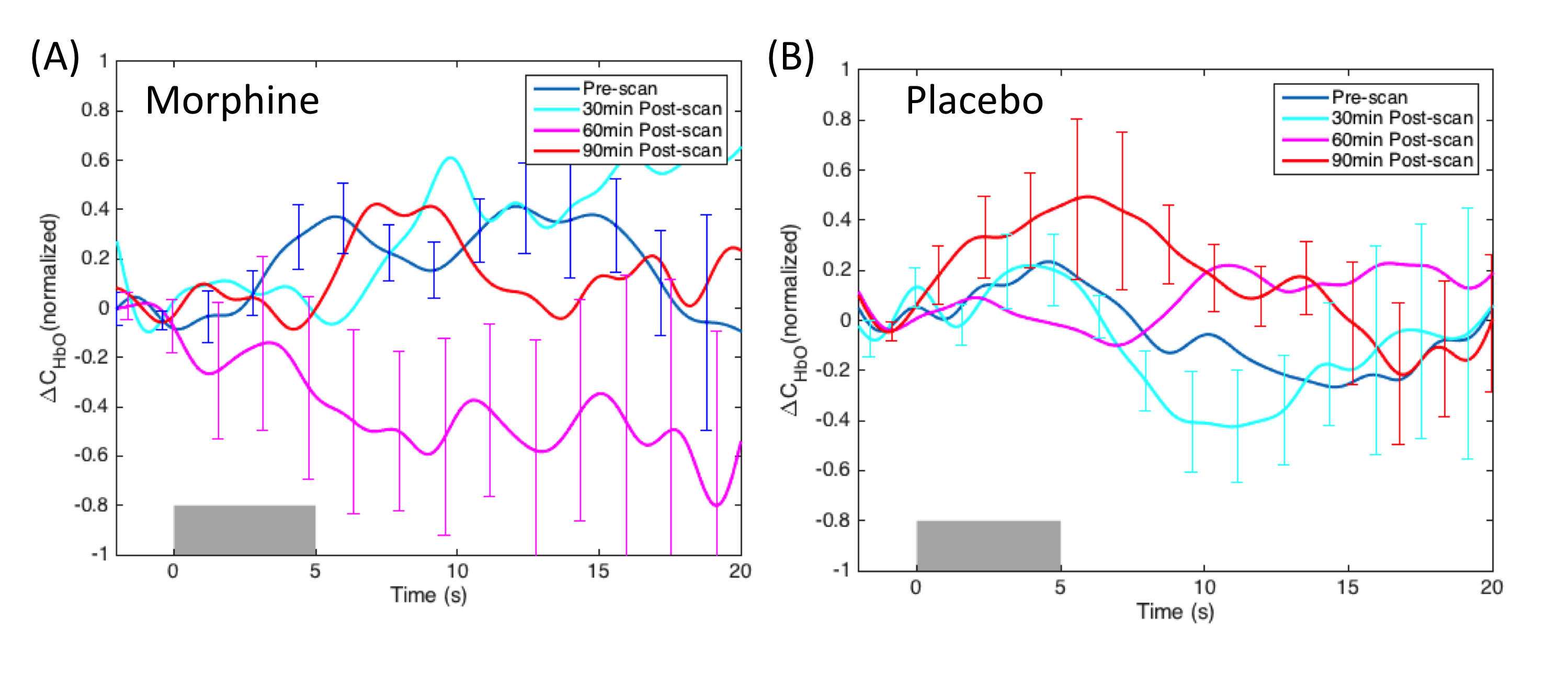


**Supplementary Figure 19.** Normalized HbO response to innocuous stimuli (VAS3) in the left lateral prefrontal cortex (averaged from channels 1-8) during morphine visits (A) and placebo visits (B). Gray bars indicate the time period when innocuous electrical stimuli were applied. All error bars show the standard error of the mean.


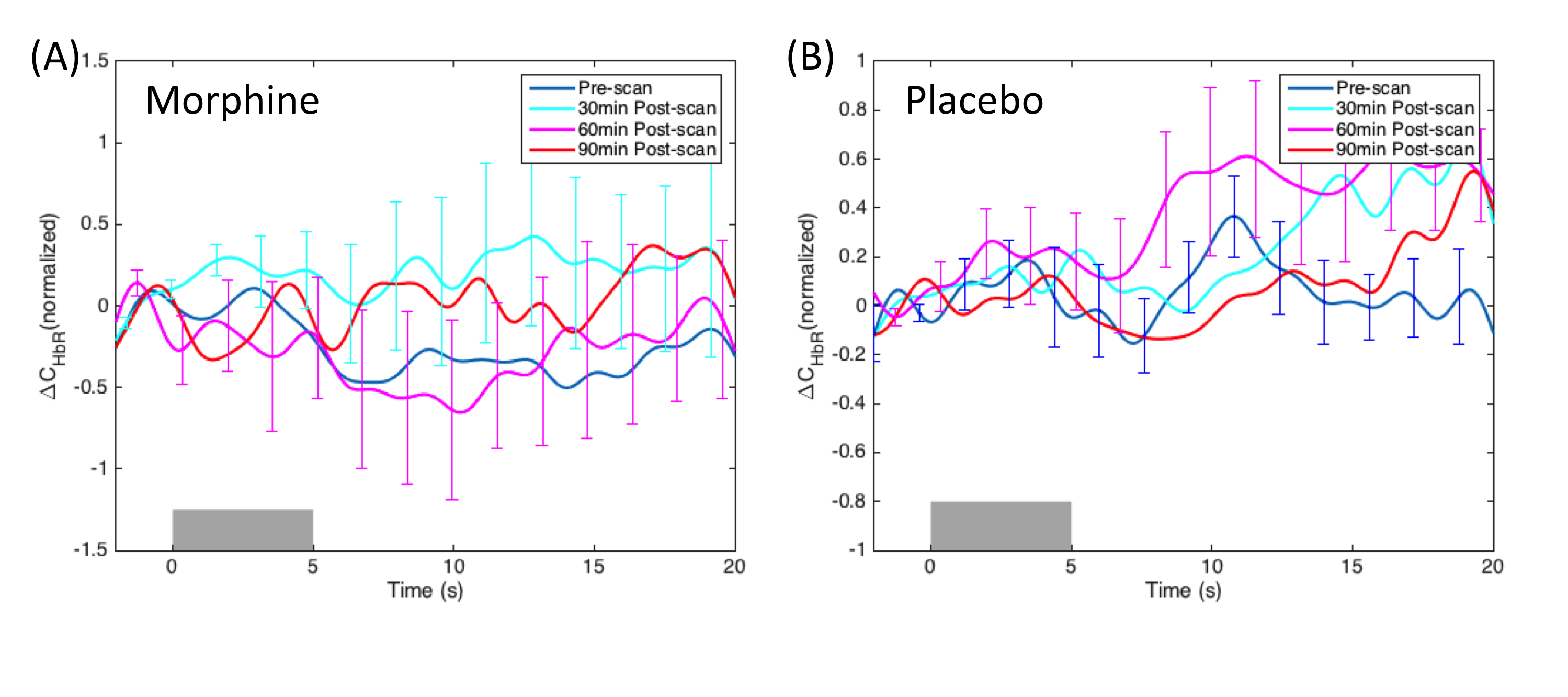


**Supplementary Figure 20.** Normalized HbR response to innocuous stimuli (VAS3) in the left lateral prefrontal cortex (averaged from channels 1-8) during morphine visits (A) and placebo visits (B). Gray bars indicate the time period when innocuous electrical stimuli were applied. All error bars show the standard error of the mean.


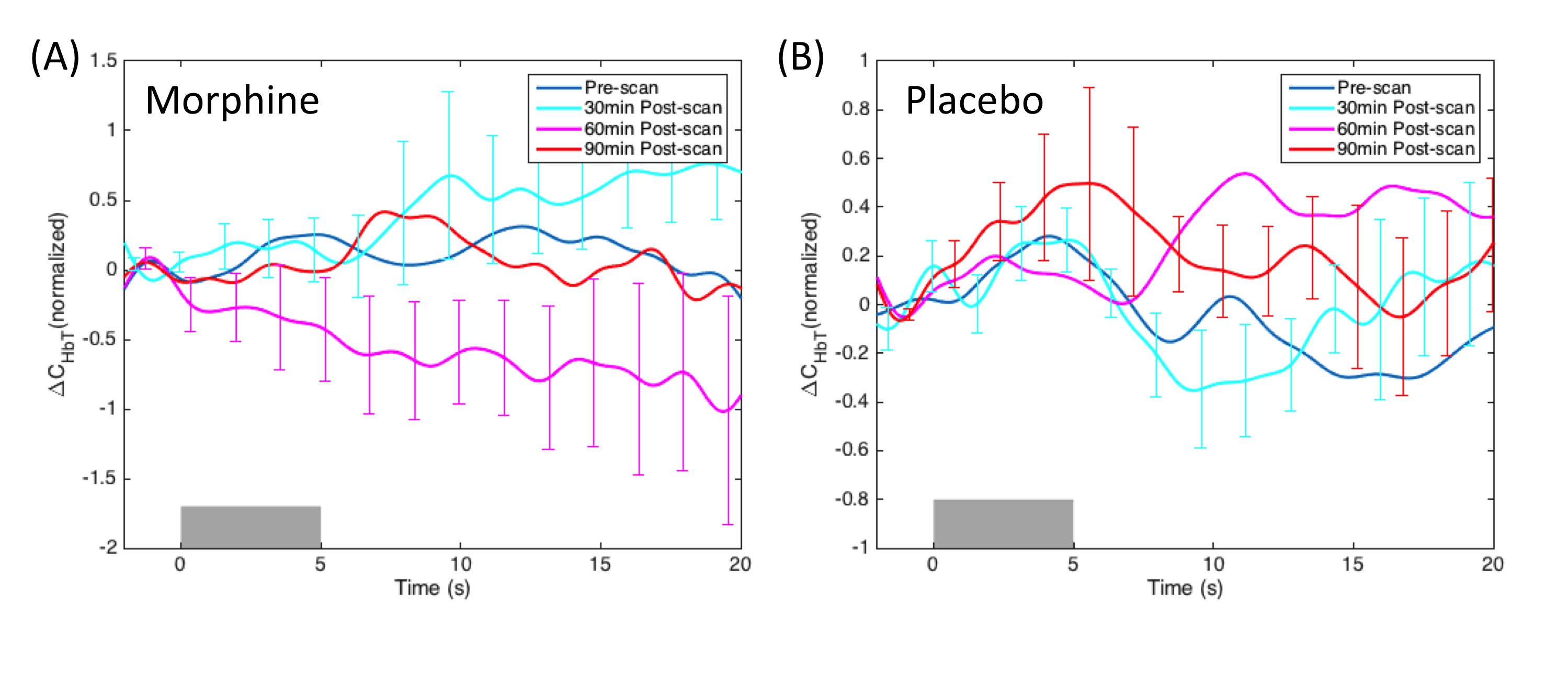


**Supplementary Figure 21.** Normalized HbT response to innocuous stimuli (VAS3) in the left lateral prefrontal cortex (averaged from channels 1-8) during morphine visits (A) and placebo visits (B). Gray bars indicate the time period when innocuous electrical stimuli were applied. All error bars show the standard error of the mean.

## Supplementary Tables

**Supplementary Table 1:** Number of noxious stimuli included in the analysis of S1 response

| #SUB | Morphine visit | | | | Placebo visit | | | |
| --- | --- | --- | --- | --- | --- | --- | --- | --- |
|  | Pre | 30min | 60min | 90min | Pre | 30min | 60min | 90min |
|  | Noxious | Noxious | Noxious | Noxious | Noxious | Noxious | Noxious | Noxious |
| 2 | 6 | 5 | 4 | 2 | 3 | 4 | 3 | 4 |
| 3 | 5 | 4 | 6 | 6 | 5 | 6 | 4 | 6 |
| 4 | 5 | 4 | 3 | 3 | 4 | 6 | 6 | 5 |
| 6 | 5 | 4 | 4 | 5 | 5 | 4 | 3 | 2 |
| 11 | 6 | 4 | 6 | 6 | 6 | 6 | 3 | 6 |
| Subtotal | 27 | 21 | 23 | 22 | 23 | 26 | 19 | 23 |
